# Base editing-derived models of human *WDR34* and *WDR60* disease alleles replicate retrograde intraflagellar transport (IFT) and hedgehog signaling defects

**DOI:** 10.1038/s42003-026-10507-2

**Published:** 2026-07-01

**Authors:** Dinu Antony, Elif Yilmaz Güleç, Anna Klawonn, Zeineb Bakey, Isabel Schüle, Gwang-Jin Kim, Toni Cathomen, Ilona Skatulla, Han G. Brunner, Sebastian J. Arnold, Miriam Schmidts

**Affiliations:** 1 Center for Pediatrics and Adolescent Medicine, University Hospital Freiburg, Faculty of Medicine, Freiburg University, Freiburg, Germany; 2 Genome Research Division, Human Genetics Department, Radboud University Medical Center and Radboud Institute for Molecular Life Sciences, Nijmegen, The Netherlands; 3 Institute for Transfusion Medicine and Gene Therapy, Medical Center-University of Freiburg, Freiburg, Germany; 4 Center for Chronic Immunodeficiency (CCI), Medical Faculty, University of Freiburg, Freiburg, Germany; 5 Department of Medical Genetics, Istanbul Medeniyet University Medical School, Kanuni Sultan Suleyman Training and Research Hospital, Istanbul, Turkey; 6 Medical Genetics Clinic, Istanbul Goztepe Prof Dr Suleyman Yalcin City Hospital, Istanbul, Turkey; 7 CIBSS - Centre for Integrative Biological Signalling Studies, University of Freiburg, Freiburg, Germany; 8 Spemann Graduate School of Biology and Medicine (SGBM), Freiburg, Germany; 9 Faculty of Biology, University of Freiburg, Freiburg, Germany; 10 Institute of Experimental and Clinical Pharmacology and Toxicology II, Faculty of Medicine, Albert-Ludwigs-University, Freiburg, Germany; 11 Maastricht University Medical Center, and GROW School of Oncology and Reproduction, Maastricht University, Maastricht, The Netherlands

**Keywords:** Development, Molecular medicine, Paediatric kidney disease

## Abstract

Cytoplasmic Dynein-2 / IFT-dynein is the only known retrograde motor for intraflagellar transport. Dysfunction of the two intermediate dynein chains WDR34 and WDR60 causes Short Rib Thoracic Dystrophy (SRTD), a human skeletal chondrodysplasias with high lethality. However, individual protein functions are incompletely understood as complete loss of function of WDR34 or WDR60 is lethal in vertebrates and individuals with SRTD carry at least one putative hypomorphic missense allele. Gene knockout is therefore not suitable to study the effect of these human missense disease alleles. Therefore, using CRISPR single base editors, we recreate three different human disease missense alleles, including a novel WDR60 variant, p.Ala968Val identified in this study. Consistent with previous findings in the dynein-2 full loss of function models and patient fibroblasts, mutant cell lines show hedgehog signaling defects as well as disturbed retrograde IFT. Transcriptome analyses reveal differentially regulated expression of genes associated with various biological processes, including regulation of the actin cytoskeleton. Further, we observe differential regulation of genes associated with Golgi intracellular transport. In addition to providing cellular model systems enabling investigations of the effect of human SRTD disease alleles, our findings indicate non-ciliary functions for WDR34 and WDR60 in addition to the established roles as components of the retrograde IFT motor complex in cilia.

## Abstract (web summary)

Base editing derived cell models shed light on the role of ciliary dynein complex components for intraflagellar transport, hedgehog signaling and differential regulation of genes associated with Golgi intracellular transport using human disease alleles.

## Introduction

**Fig. 1:**
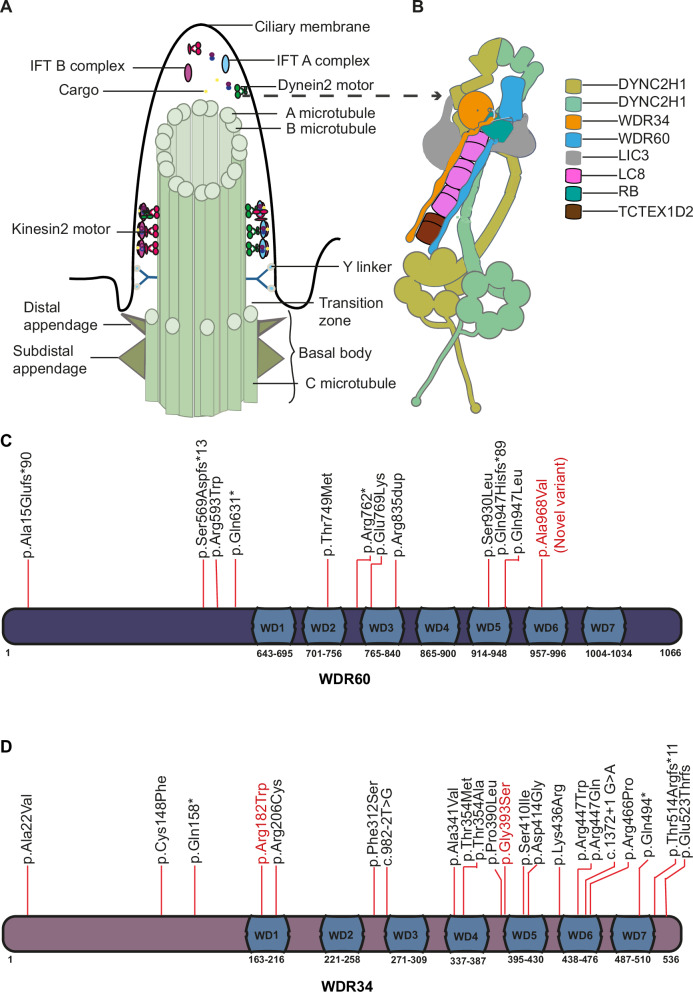
Primary cilia ultrastructure, intraflagellar transport, and cytoplasmic dynein-2 composition and function. **A** Kinesin-2 motor powers the anterograde transport, and a dynein-2 motor powers the retrograde cargo traffic. IFT-A and IFT-B complexes attach to the motor complexes and serve as cargo adapters. **B** Schematic of Dynein-2 motor structure. The two intermediate chains, WDR34 and WDR60, bind to the heavy chains as a heterodimeric complex with light chain binding mediated by the N-terminal domains, adapted from refs. ^47,66^. **C** WDR60 protein structure. Known human SRTD disease alleles are indicated; the novel, not previously described disease variant reported in this paper and recreated in cilia–APEX–IMCD3 cells is marked in red. **D** WDR34 protein structure. Known human SRTD disease alleles; the variants recreated in cilia-APEX-IMCD3 cells are marked in red.

Dyneins are minus-end-directed multiprotein motor complexes consisting of heavy-, intermediate-, light intermediate-, and light-chains. Powered by ATP, dynein motors transport various cargoes, vesicles, and organelles along microtubules. Three different types of dynein motors can be distinguished: axonemal dyneins, dynein-2 (intraflagellar Transport (IFT) dynein), and cytoplasmic dynein^1^. While cytoplasmic dynein is essential for retrograde transport along cytosolic microtubules and mitotic spindle arrangement^2^, axonemal dyneins and IFT dynein are solely found in cilia^1^. Axonemal dyneins are essential for ciliary motility, and dysfunction of axonemal dynein motor proteins causes primary ciliary dyskinesia (PCD) (MIM #244400), a chronic respiratory disease characterized by recurrent respiratory infections, laterality defects, and subfertility^3^. Dynein-2 is the only known ciliary retrograde motor complex and controls the transport of cargo from the tip of the cilia back to the base (retrograde IFT) (Fig. 1A).

Dynein-2 dysfunction results in a ciliopathy phenotype known as “short rib thoracic dysplasia/dystrophy (SRTD)” in humans. Shortened ribs and long bones resulting in a constricted thoracic cage, pulmonary hypoplasia, and polydactyly are hallmark features of SRTD. Radiologically, so-called handlebar clavicles, a trident appearance of the acetabular roof, and cone-shaped epiphyses can be observed. Individuals with short-rib polydactyly syndromes (SRPS; MIM #611263, MIM #613091, MIM #263520, MIM #269860, and MIM #614091) hereby represent the severe end of the phenotypic spectrum, with affected individuals dying prenatally or soon after birth due to cardiorespiratory failure. Jeune syndrome (Asphyxiating Thoracic dystrophy; JATD, MIM 208500) patients present the milder end of the phenotypic spectrum and often survive beyond the neonatal period^4,5^. While structural heart defects, cystic kidneys, and CNS malformations have been described in a subset of SRPS cases, this is not usually the case for JATD individuals^4,5^.

Recessively-inherited mutations causing SRTD have been identified in most genes known to encode for dynein-2 subunits, including the heavy chain *DYNC2H1*^6^, the two intermediate chains *WDR34*^7,8^ and *WDR60*^9^, the light intermediate chain *DYNC2LI1*^10^, and the light chain *TCTEX1D2*^11^. No mutations have been identified in other light chains to date. A schematic of dynein-2 structure is shown in Fig. 1B. *WDR60* variants have been recently reported in a family with retinal degeneration and polydactly but without any skeletal dysplasia phenotype^12^, while *WDR34* variants have been additionally identified in a case with non-syndromic rod cone dystrophy (RCD)^13^. WDR60 and WDR34 protein structures and localization of published disease-causing variants are shown in Fig. 1C, D, respectively. Interestingly, all human SRTD cases reported to date carry at least one presumably hypomorphic (mostly missense) allele^7–9,14^. This is in line with the observation that complete knockout of *Dync2h1*, *Wdr34,* or *Wdr60* is lethal around mid-gestation in mice^15–17^, and results in severe ciliogenesis defects also in vitro^18^. This is not observed in *WDR34* mutant patient fibroblasts or WDR60 mutant JATD fibroblasts, while WDR60 mutant SRPS fibroblasts have been found to exhibit lower ciliation rates than controls^7,9^.

Recreating hypomorphic human disease alleles is better suited to study the effect of patient missense alleles than gene knockout, as these alleles retain some function and do not affect the ciliary structure to the same extent as a complete knockout. Especially, it allows avoiding the secondary effect of severely shortened cilia, as observed for example for WDR34 complete loss of function as shown by Tsurumi et al. (2019); Vuolo et al. (2018), and Wu et al. (2017). Ciliary truncation is not observed in primary cells from human patients and could result in secondary effects unrelated to human disease alleles^17–19^.

We therefore used CRISPR–Cas9-based cytosine base editing to generate mutant cell lines carrying hypomorophic human disease alleles homozygously, including a novel WDR60 disease allele, and detected retrograde IFT defects as well as disturbed hedgehog signaling in all mutant clones. GO term analyses of mutant versus control ciliated cells revealed upregulation of G-protein-coupled receptor signaling as well as differentially regulated transcription of genes associated with extracellular matrix composition, endochondral bone growth, and chondrocyte development. Strikingly, we also observed changes in the expression of many Golgi-associated genes and found delayed Golgi recovery induction of monensin-mediated stress in mutants compared to controls. In light of the concordantly observed downregulation of *Rab6b* in all mutant clones, this could indicate that impaired Golgi function and Golgi-associated protein transport may contribute to the pathomechanism underlying the patient phenotypes caused by the WDR34 and WDR60 dysfunction.

## Results

### Identification of a novel disease-causing WDR60 allele using exome sequencing

**Fig. 2:**
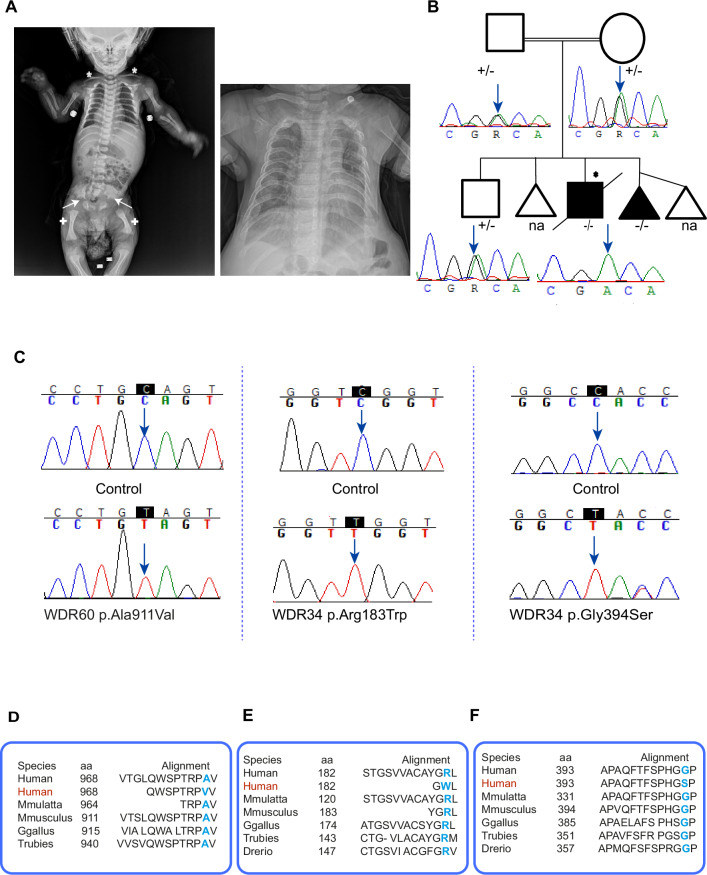
Segregation analysis of the novel WDR60 c.2903 C > T, p.Ala968Val variant identified and replication of human disease alleles using CRISPR base editing. **A** Radiographs of the index case showing typical clinical hallmarks of SRTD, including shortened ribs, a narrow thorax, shortened long bones (indicated by +), handlebar clavicles (indicated by *), and acetabular spurs (arrows). **B** Sanger sequencing confirmed that parents and the unaffected sibling carry the allele heterozygously while the affected index (asterisk) carries the variant in a homozygous state, confirming an autosomal recessive inheritance pattern. **C** Genotypes of homozygously edited clones displaying human disease alleles. Chromatograms depict the single-base changes introduced (arrowheads). Note, for WDR34p.Gly394Ser reverse primer is used for Sanger sequencing. **D**–**F** Cross-species conservation of amino acid positions affected by introduced human disease alleles.

Individual SI_36, offspring of consanguineous Turkish parents, was referred to us for genetic analysis from a local genetics clinic, presenting with clinical signs of Short rib thoracic dysplasia (SRTD) including short ribs and long bones, brachydactyly, a narrow thorax, handlebar clavicles, acetabular spines on pelvis x-ray examinations as well as recurrent lower respiratory tract infections and lethal cardiorespiratory insufficiency at the age of 18 months (Fig. 2A, a detailed description of the clinical course can be found in the Supplementary Information File). Exome sequencing revealed a novel homozygous *WDR60* variant (NM_018051.5, c. 2903 C > T, p.Ala968Val), not previously described as disease-causing in humans and not reported in gnomad (https://gnomad.broadinstitute.org/), indicating it is a very rare allele (Table 1). The variant is located at the WD domain of the protein (Fig. 1C) and segregated in an autosomal recessive fashion in the family (Fig. 2B). The variant is predicted to be disease causing by mutation taster (score obtained: 64; the prediction score ranges from 0 to 215; https://www.mutationtaster.org/) and probably damaging by polyphen-2 (0.99; the prediction score ranges from 0 to 1; http://genetics.bwh.harvard.edu/pph2/).

**Table 1:** Published *WDR60* variants and associated phenotypes

Case	WDR60 SRTD variants in human	Skeletal phenotype	Visceral organ phenotype	Other organ involvement	References
1	p.Ser569Aspfs*13, p.Thr749Met Compound heterozygous	Short femora, a narrow chest, preaxial polydactyly,	Small clinically insignificant ventricular septal defect	None	^9^
2	p.Gln631* & p.Thr749Met Compound heterozygous	Short long bones, severe shortening of long bones with bowed femurs, macrocephaly, short ribs, postaxial polydactyly, syndactyly, brachydactyly, acetubular spurs,	Pancreatic fibrosis, mild dilatation of renal tubules, and an enlarged liver with ductal plate malformation	Ambiguous genitalia,	^9^
3.	p.Gln631* & p.Thr749Met Compound heterozygous Sibling of case 2	Short ribs, short bowed limbs, brachydactyly, conical epiphyses, hypoplastic trabecular, depressed nasal bridge,	Ventricular septal defect (VSD), focal cystic changes in the kidneys, prominent bile duct plates, pulmonary hypoplasia,	None	
4.	p.Gln947Leu Homozygous	The chest is very narrow and bell-shaped. Short arms and legs, hands showed hypoplastic phalanges with cone-shaped epiphyses, syndactyly, narrow, bell-shaped chest, elevated horizontal clavicles, horizontal short ribs with enlarged costochondral junction, mild pattern of short-limbed dwarfism with reduced length of the long bones, bowing of the femora, humeri with irregular metaphyses, and shortened iliac bone with trident-shaped acetabular roof	Hypoplastic lungs, mild liver disease	None	^64^
5 + 6 (siblings)	p.Ala15Glufs*90 Homozygous	Postaxial polydactyly, mild syndactyly, short stature, relatively thin long tubular bones (Phenotype is similar for both siblings)	Mild hepatomegaly	Slowly progressive bilateral pigmentary retinal dystrophy with night blindness followed by loss of peripheral visual acuity	^12^
7	p.Arg593Trp Homozygous	JATD (Details of the individual cases were not described). Used the following phenotype to assign as JATD: long, narrow chests, moderately short ribs, handlebar clavicles, trident acetabulae, and short extremities. Brachydactyly was typically present with polydactyly as a variable feature. The ends of the long bones had metaphyseal abnormalities that were either smooth or had lateral spikes. The overall skeletal findings were somewhat similar to all types of SRPS, but with less severe features.	None	None	^14^
8	p.Glu769Lys Homozygous	JATD (Details of the individual cases were not described). Assigned as JATD using the phenotypes described in case 7			
9	p.Glu769Lys & p.Gln947Hisfs*89 Compound heterozygous	SRPS III, Polydactyly was present (Details of the individual cases were not described). Used the following phenotypes to assign as SRPS III Similar to but less severe than SRPS type I, with small chests and short limbs. Radiographs showed short, horizontal ribs and long bones with lateral metaphyseal spikes. Distal extremities were short and poorly mineralized, especially the carpal and distal phalangeal bones. Polydactyly was a variable feature. Small iliac bones with a trident pelvis and poorly formed scapulae were common, as there were multiple craniofacial anomalies.	None	None	^14^
10	p.Arg835dup & p.Arg762* Compound heterozygous	SRPS III, Polydactyly was present (Details of the individual cases were not described). Assigned as SRPS III using the phenotypes described in case 9	None	None	^14^
11	p.Ser930Leu	Disproportional limb shortening, a conspicuous small chest, and short extremities with brachydactyly, accompanied by deformed teeth	Chronic kidney disease (stage 5)	Bilateral retinitis pigmentosa	^65^
12	p.Ala968Val (Novel variant)	Narrow thoracic cage, predominantly rhizomelic short extremities (femur, humerus, tibia), mild angulation on the humerus, short hands and feet, brachydactyly, syndactyly	Hepatosplenomegaly, hydronephrosis of the left kidney, relative cardiomegaly, right aberrant subclavian artery	None	Novel variant identified in this study

### Recreation of human WDR34 and WDR60 missense alleles using CRISPR base editing

To investigate the functional effects of the novel *WDR60* allele as well as previously published *WDR34* SRTD patient missense alleles, we proceeded to perform CRISPR base editing to recreate human disease causing mutations in Cilia–APEX–IMCD3 cells^20^ (Supplementary Fig. 1). We were able to recreate two *Wdr34* missense alleles homozygously: *Wdr34* NM_001008498.2 (ENSMUST00000113711.3) c.547 C > T, p.Arg183Trp and c.1180 G > A, p.Gly394Ser (in human: *WDR34* NM_052844.3:c.544 C > T, p.Arg182Trp and c.1177 G > A p.Gly393Ser) as well as the novel *WDR60* allele we identified in proband SI_36.1 (case 12 in Table 1) (human c. 2903 C > T, p.Ala968Val): mouse *Wdr60* NM_146039.3 (ENSMUST00000039349.8), c.2732 C > T p.Ala911Val. When recreating the intended C–T change in *Wdr60*, the cytidine deaminase created an additional heterozygous C-to-T change 7 bases downstream of the targeted base, resulting in a heterozygous synonymous change (c.2739 C > T, p.Phe913Phe), hence likely without functional consequences. Likewise, an unintended additional heterozygous change (*Wdr34* c.1178 G > A) causing a glycine to aspartic acid change at position 393 was created in the *Wdr34* c.1180 G > A, p.Gly394Ser clone (Fig. 2C). Base editing efficiencies were 0.66% for *Wdr60*, c.2732 C > T, p.Ala911Val, 2% for *Wdr34* c.547 > T, p.Arg183Trp and 13% for *Wdr34* c.1180 G > A, p.Gly394Ser. The affected protein positions are evolutionarily well conserved across organisms (Fig. 2D–F).

### Retrograde IFT trafficking defects and perturbed hedgehog signaling in Wdr60 and Wdr34 mutant clones

**Fig. 3:**
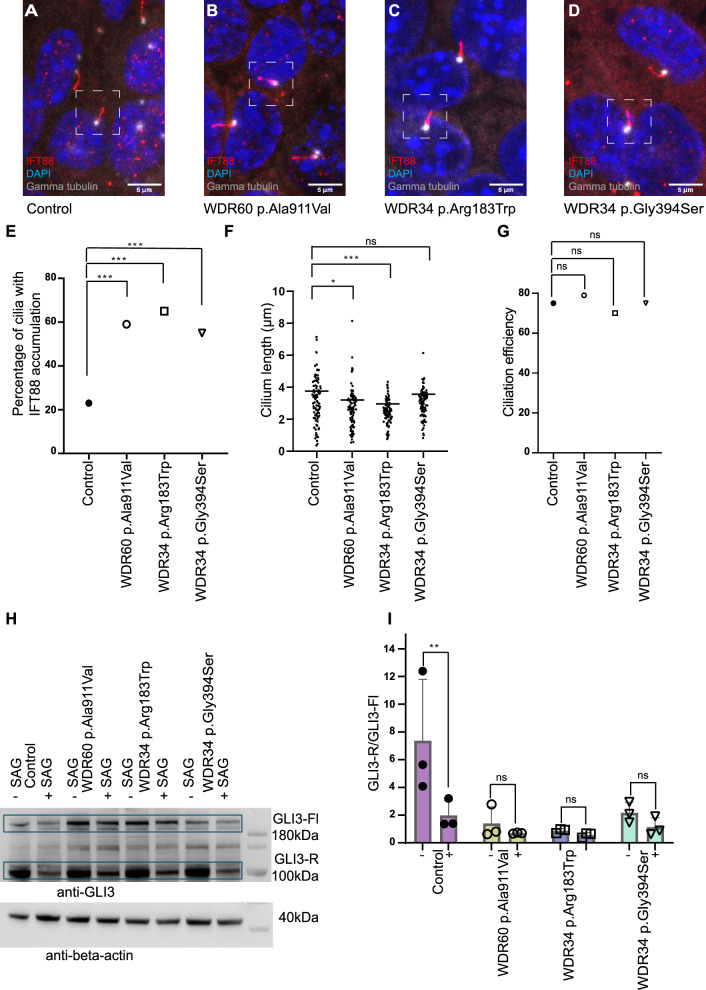
Cellular phenotyping of generated mutants reveals no overt ciliogenesis defects but defective retrograde IFT and disturbed hedgehog signaling. **A**–**D** Immunofluorescence analysis of the IFT-B component IFT88 reveals accumulation at the ciliary tip, indicative of defective retrograde IFT. Ciliation was induced by serum starvation. In immunofluorescence analysis, the ciliary base is marked by gamma tubulin (Gray), and IFT88 is shown in red. Images were taken using a Zeiss LSM NLO inverted microscope. Scale bars: 5 µm. **E** Quantification of IFT88 accumulation at the ciliary tip in mutant versus control cells. Dynein-2 intermediate chain mutant clones showing significant accumulation of IFT88 at the tip of cilia compared to control cells. (Fisher’s exact test, *n* = 130 cells analyzed per genotype). **F** Cilia length distribution in dynein-2 intermediate chain mutants compared to controls (student’s *t*-test, *n* = 100 cells per genotype analyzed, line at mean value). WDR60p.Ala911Val and WDR34p.Arg183Trp mutant cells displayed a significant reduction in cilia length compared to controls, while no significant difference was observed for WDR34p.Gly394Ser mutant cells. **G** Ciliation efficiency of dynein-2, intermediate chain mutants appeared similar to controls, with at least 75% of ciliation detected in all clones (*n* = 100 cells per genotype analyzed, Fisher’s exact test). **H** Western blot analysis revealed increased GLI3 full-length (GLI3-Fl) levels in all dynein-2 intermediate chain mutants compared to controls with and without addition of the hedgehog inducer SAG, while baseline GLI3 repressor (GLI3-R) levels appeared unchanged. **I** Densitometry analysis of western blot bands and calculation of GLI3 repressor to GLI3 full length ratios amounts before (first bar) and after SAG treatment (second bar), revealed a significant reduction in GLI3 repressor to GLI3 full length ratio after SAG treatment in control but not mutant cells (two-way annova with Bonferroni’s multiple comparisons test).

To analyze potential ciliary defects in the three created dynein-2 intermediate chain mutant clones, we proceeded to investigate retrograde IFT visualized by immunofluorescence analysis of the IFT-B component IFT88. All three dynein-2 intermediate chain mutant clones showed significant accumulation of IFT88 at the ciliary tip compared to the cilia of control cells (Fig. 3A–E). Although some cilia displayed IFT88 accumulation at the base, our focus was on IFT88 accumulation at the tip. This allows us to compare our findings with those previously published in patient fibroblasts, where accumulation at the base was not assessed. WDR60p.Ala911Val and WDR34p.Arg183Trp mutant cells exhibited a small but statistically significant reduction in cilia length compared to control cells, while no statistically significant difference was observed for WDR34p.Gly394Ser mutant cells for which the ciliary length was similar to controls (Fig. 3F). The percentage of ciliated cells was similar among mutants and controls (Fig. 3G).

All mutant clones showed perturbed hedgehog signaling in comparison to controls. While addition of the hedgehog signaling agonist SAG resulted in a significant reduction of the GLI3 repressor (GLI3-R) to GLI3 full length (GLI3-Fl) ratio in control cells as expected, this was not observed in WDR34p.Arg183Trp, WDR60p.Ala911Val and WDR34p.Gly394Ser mutant cells (Fig. 3H, I, Supplementary Fig. 2). However, in line with previously reported findings in *Wdr34* mutant mouse embryos^17^, full-length GLI3 levels were higher in all dynein-2 intermediate chain mutants compared to control cells at the basal state (Fig. 3H and Supplementary Fig. 2), likely accounting for the reduced effect of Gli3R/Gli3fl ratio after SAG addition.

### WDR34 and WDR60 human disease alleles influence gene transcription and suggest dynein-2 intermediate chain dysfunction affects Golgi protein transport

**Fig. 4:**
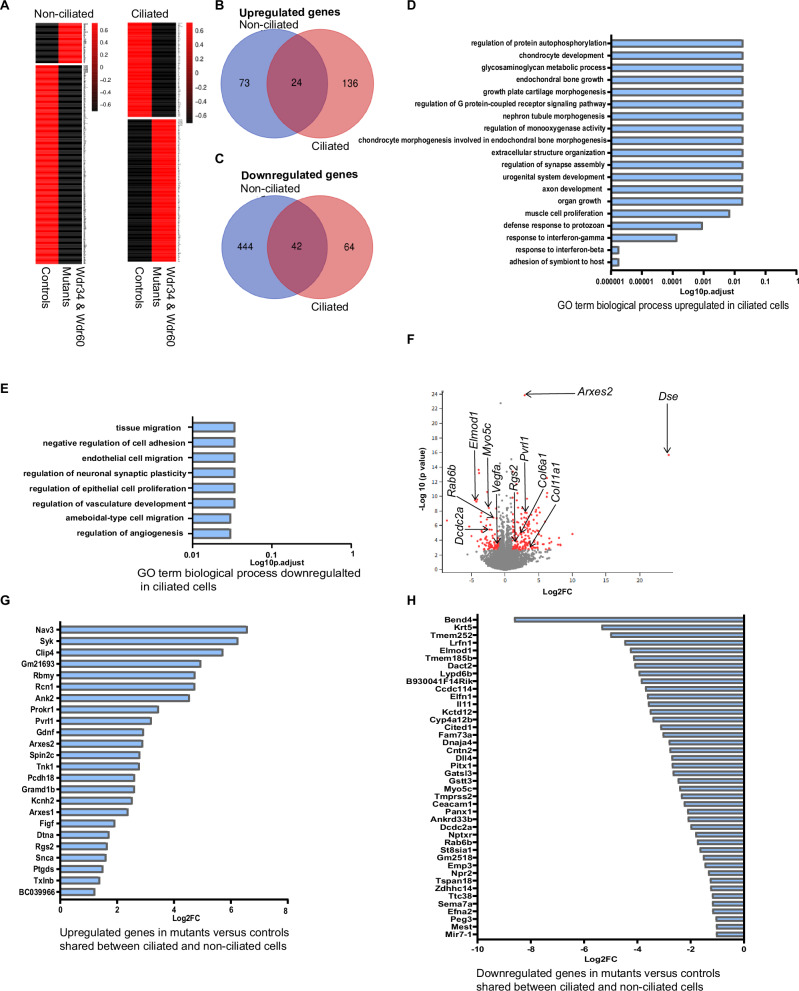
Differential gene expression between dynein-2 intermediate chain mutants and controls. **A** Heat Map analyses of transcriptome analyses showing a higher number of differentially expressed genes in non-ciliated cells compared to ciliated cells, with more genes downregulated in mutants compared to controls. **B** Venn diagram showing upregulated genes in mutants versus controls, of which 24 genes are commonly upregulated in both ciliated and non-ciliated cells. **C** Venn diagram showing downregulated genes in mutants versus controls, with 42 genes commonly downregulated in both ciliated and non-ciliated cells. **D** GO term analyses of genes upregulated in mutants versus controls in ciliated cells. **E** GO term analyses of genes downregulated in mutants versus controls in ciliated cells. **F** Volcano plot showing differentially expressed genes of ciliated mutants versus controls. **G** Upregulated genes in mutants versus controls shared between ciliated and non-ciliated cells (*p* < 0.05 and log2FC ≥ 1). **H** Downregulated genes in mutant versus controls shared between ciliated and non-ciliated cells (*p* < 0.05 and log2FC ≤ −1).

To investigate the effects on gene expression resulting from WDR34 and WDR60 patient alleles, we next performed RNA sequencing of the three mutant clones and four different controls. To dissect putative functions related to ciliogenesis, we chose to analyze both ciliated and non-ciliated cells. To minimize clonal effects and false positive hits, we investigated all three ciliated mutant clones versus three different control clones, while WDR34 p.Gly394Ser was not included in the non-ciliated clone analysis, as this mutant was obtained only after the RNA sequencing for non-ciliated cells was completed. While WDR34 and WDR60 have unique functions within the dynein-2 complex^18,21^, dysfunction results in the same human phenotype, indicating similar downstream effects, hence justifying a combined analysis to identify the pathomechanism underlying JATD/SRPS in humans and to reduce the number of false-positive hits. We observed a higher number of differentially expressed genes between mutants and controls in non-ciliated cells compared to ciliated cells, with a greater number of genes downregulated in mutants compared to controls in non-ciliated cells. In contrast, more genes appeared upregulated in ciliated mutants compared to ciliated controls (Fig. 4A–C). Potentially, WDR34p.Gly394Ser not being included in the transcriptomics analysis of non-ciliated cells could contribute to these differences.

We started by analyzing ciliated cells, given the established role of WDR34 and WDR60 for retrograde IFT and cilia function. Gene ontology (GO) term biological process pathway analyses for genes upregulated in mutant ciliated cells compared to controls revealed G-protein-coupled receptor signaling as well as several terms related to skeletal development, such as endochondral bone growth, chondrocyte development, and extracellular structure organization (Fig. 4D). Differentially expressed genes encoding for proteins associated with G-protein-related signaling included GTPase activators *Rgs2* and *Rgs9*^22^, as well as *Snca*. RGS2 can inhibit COPI (Coat protein I) binding to the Golgi and affect Golgi-mediated intracellular transport^23^. *Snca* encodes alpha-synuclein, a substrate of G protein-coupled receptor kinases. Synuclein accumulation has been associated with Parkinson’s disease, and overexpression of *Snca* in rat ventral midbrain cultures caused reduced neurite length and increased Golgi fragmentation^24^. Genes associated with extracellular structure organization, endochondral bone growth, and adhesion to symbiont host included *Col6a1, Col6a2, Col11a1, Col16a1*, and *Pvrl1* (NECTIN-1). *Pvrl1* encodes for an actin-binding cell adhesion molecule^25^ (Supplementary Table 1). Western blot analysis confirmed the upregulation of PVRL1 in mutant clones compared to controls (Supplementary Fig. 3).

Downregulated pathways and processes revealed by GO term analysis included angiogenesis/vasculature development, endothelial cell migration, tissue migration, regulation of epithelial cell proliferation, negative regulation of cell adhesion and regulation of neuronal synaptic plasticity (Fig. 4E). Among the single genes differentially regulated between ciliated mutants and controls was *Vegfa* (vascular endothelial growth factor-A), playing an important role in angiogenesis^26^ (Fig. 4E, F). Go term biological process pathway analyses and volcano plot of individual mutant clones in comparison with three controls in ciliated cells are also indicated in (Supplementary Figs. 4–12).

To complement our analyses of all ciliated mutants versus all controls analyzed together, we also performed analyses of single mutant genotypes, the different WDR34 and WDR60 mutant clones separately, versus control, and searched for potential shared mechanisms or pathways using GO analysis comparisons. This revealed few shared processes between all three genotypes, mainly due to the low number of GO terms for WDR34Arg183Trp. Such shared processes included “synapse assembly/ organization” (GO:0007416/ GO:0051963; including *Arhgef9, Nfatc4, Cbln1, Magi2, Ntn1, Adgre5, Adgrb2, Flrt1, Dnm3, Nptxr, Lrfn1,* and *Epha7*) and “positive regulation of vasculature development” (GO:1904018, including *Gper1, Fgf1, Vegfd, Efnb2, Pdgfd, Hyal1, Aggf1, Ctsh, C3, Sema5a, Igf2, Cd34*) / angiogenesis (GO:0045766, *Ptn/Ccl2, Vegfc, Dcn, Enpp2, Hgf, Cd36, Ptgis, Bmper, Cxcr4, Igf2, Aqp1, Vegfd, Agt, Itgb3, Gata2, Flt1, Itgb8, Stat1, Sema4a, Vegfa, F3, Ghrl, Hspb1, Hmga2, Tert, Ccbe1, Adam12, Ceacam1, Dll4/Cd34*) (Supplementary Figs. 4–9, Supplementary Data 1–3).

WDR60 and WDR34p.Gly394Ser mutant versus control analyses also shared the GO terms renal system development (GO:0072001; *Gdnf, Fgf1/Efnb2, Irx1, Pdgfd, C1galt1, Tgfb2, Col4a1, Foxd1, Gcnt1, Wnt6, Dact2, Cited1, Epha7*), branching morphogenesis related terms such as “morphogenesis of a branching structure” (GO:0001763), “axon development” (GO:0061564*; Gdnf, Nrep, Mapt, Ust, Efnb2 Braf, Map1b, Plxnd1, Lhx2, Ntn1, Tgfb2, Foxd1, Sema5a, Map2, Tnc, Artn, Gas1/, Epha7, Alcam*) and “negative regulation of growth” (GO:0045926; *Fbp1, Hyal1/, Ntn1, Stc2, Tnk1, Tgfb2, Sema5a, Map2, Cyp27b1, Gas1, Lta, Epha7, Enpp1*). While no single shared GO term related to skeletal/cartilage development came up in this analysis, “hyaluronan metabolic process” (GO:0030212, *Il15, Cd44/Itih4, Cemip*) was observed as significant term in WDR34 Arg183Trp versus controls, while “cartilage development” (GO GO:0051216; Nov, *Mef2c, Sulf2, Col11a1, Prrx1, Col6a2, Col6a1, Rarb, Adamts12, Mycn, Matn2, Axin2, Itgb8, Hoxd3, Msx1, Col7a1, Col2a1, Bmp7*) and “chondrocyte development” (GO:0002063; *Sulf2, Col11a1, Col6a2, Col6a1, Matn2, Axin2, Col7a1*) were significant terms in WDR34 p.Gly394Ser versus control analyses. In WDR60 mutant versus control cells, no chondrocyte, cartilage, or skeletal development-associated term came up in GO analysis. Given that human individuals affected by WDR34 and WDR60 share a phenotypically indistinguishable skeletal phenotype of comparative severity, this was somewhat surprising and could stem from genotype-independent inter-clonal variability masking common mechanisms.

Many GO terms were unique to single genotypes versus controls, with the GO term “dichotomous subdivision of an epithelial terminal unit” (*Celsr1*, *Plxnd1*, *Ctsh*, and *Foxd1)* being one of the most significantly downregulated terms in WDR60 mutant versus control cells only (Supplementary Fig. 5, Supplementary Data 1). *Celsr1* (cadherin EGF LAG seven-pass G-type receptor 1) encodes a protein playing an important role in planar cell polarity (PCP)^27^. Interestingly, in line with our finding, Yan et al. have observed lower levels of *Celsr1* expression in *Wdr34* but not *Wdr60* mutant mice and concluded that WDR34 could play a more important role in planar cell polarity than WDR60^28^. The differentially expressed genes in the single mutant clones are provided in the Supplementary Data 4–6.

In order to also identify cilia-independent functions of WDR34 and WDR60, we next searched for genes for which expression levels were similarly influenced in ciliated and non-ciliated cells. GO term analysis revealed downregulation of biological processes important for cell and tissue migration (Supplementary Fig. 13). We identified 66 differentially regulated genes between mutants and controls in both non-ciliated and ciliated cells, of which 24 were upregulated, and 42 were downregulated (Fig. 4B, C, G, H). We would also like to emphasize that the differences in gene expression between cells grown with or without serum cannot be directly attributed to the presence or absence of a cilium, but may be the result of serum starvation, which is used to induce cilia formation. Amongst those, we observed downregulation of *Dcdc2*, encoding for a protein containing 2 doublecortin peptide domains, localizing to primary cilia and playing a role in tubulin binding, microtubule polymerization, and Wnt signaling regulation^29^. We further found that several differentially downregulated genes encode for Golgi-associated proteins and proteins involved in vesicle trafficking, including *Rab6b*^30^, the ARF GAP *Elmod1*^31^, which has also been linked to protein transport from the Golgi to the cilium^31^, *Myo5c*^32^ and *Zdhhc14*^33^ (Fig. 4G, H). While GO term analyses did not reveal enrichment of G-protein-coupled receptor signaling, we detected upregulation of two G-protein signaling pathway-associated genes *Rgs2* and *Snca*. Likewise, *Pvrl1* was found to be upregulated in both non-ciliated cells as well as ciliated cells, while we did not observe an enrichment of extracellular structure or cytoskeleton genes in general.

**Fig. 5:**
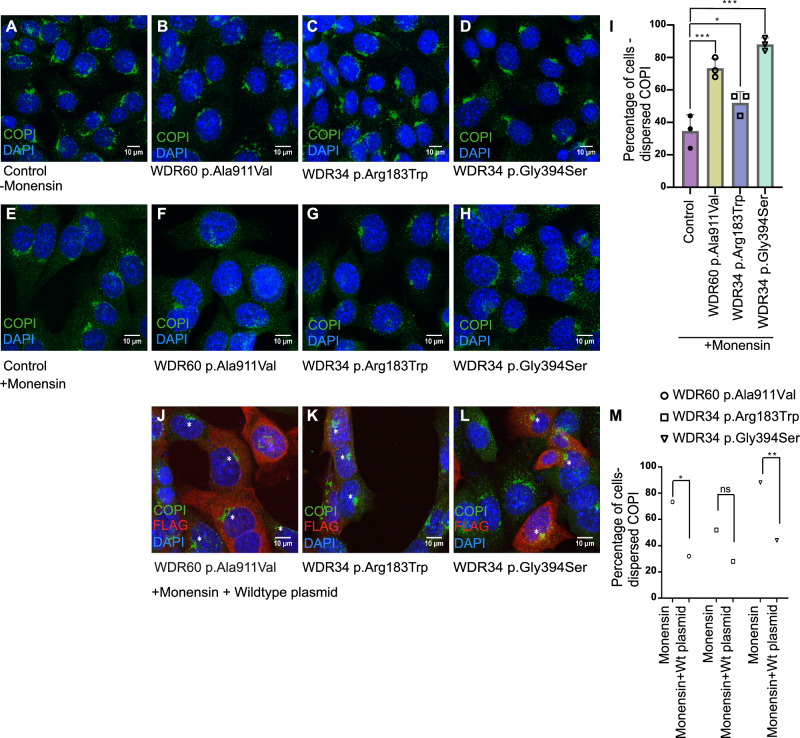
Impaired COPI-Golgi recovery after Golgi stress in mutant versus control clones. **A**–**D** COPI immunofluorescence analyses reveal no overt difference between mutant and controls under normal conditions. **E**–**H** Monensin treatment followed by washout reveals delayed recovery of the COPI-positive compartment in mutants compared to controls. **I** Quantification of immunofluorescence results obtained by calculating the percentage of cells with dispersed COPI compartment after the monensin stress assay in mutants versus controls (*n* = 25 cells per genotype analyzed, one-way ANOVA with Dunnett’s multiple comparisons test, error bar depicts SD between three independent experiments). **J**–**M** Overexpression of Flag-tagged wildtype WDR34 or WDR60 (in red) in corresponding mutant clones partially rescues the dispersed COPI compartment phenotype in the mutants resulting from monensin treatment (*p < 0.05, **p < 0.01; ***p < 0.005, scale bars: 10 µm in all images). **M** Quantification of cells with dispersed COPI compartments after monensin treatment, displaying partial recovery in all three mutants after expression of wild-type protein. Overexpression of wildtype WDR60 in WDR60 p.Ala911Val cells, as well as overexpression of wildtype WDR34 in WDR34 p.Gly394Ser mutant cells, resulted in statistically significant recovery of the dispersed COPI compartment, while statistical significance was not reached in WDR34 p.Arg183Trp cells despite visible reduction of COPI dispersion (*n* = 25 cells per genotype analyzed, Fisher’s exact test).

To further investigate putative Golgi transport defects in dynein-2 intermediate chain mutant cells, we first performed an immunofluorescence analysis of COPI vesicles in mutant cells versus controls at the basal state. This did not reveal obvious differences (Fig. 5A–D). Assuming that mutant cells may exhibit only mild defects due to the hypomorphic nature of the mutations, potentially only detectable under stress conditions, we proceeded to treat the cells with monensin, an agent causing dispersal of the Golgi apparatus, and then observed restoration of the Golgi structure after re-application of standard cell culture medium^34^. This revealed a delayed/impaired Golgi recovery for all three dynein-2 intermediate chain mutants compared to controls (Fig. 5E–I). Overexpression of GLI3 full-length in control cells did not affect the COPI vesicles (Supplementary Fig. 14I–II), indicating the Golgi transport defect observed in WDR60 and WDR34 mutant clones is due to the dysfunction of WDR34 and WDR60 proteins. Overexpression of wild-type WDR34 or WDR60 in the corresponding mutant cell lines partially rescued the delayed/impaired Golgi recovery after monensin treatment, with WDR60p.Ala911Val and WDR34p.Gly394Ser mutant clones displaying statistically significant recovery while clearly visible improvement of the golgi phenotype in WDR34p.Arg183Trp mutant cells did not quite reach statistical significance (Fig. 5J–M).

## Discussion

The hypomorphic nature of human disease alleles in dynein-2 components suggests that knockout models may be less suitable for functional studies. Using CRISPR-based editing, we were able to replicate three human disease alleles in cilia-Apex-IMCD3 cells. However, editing efficiency using a BE4-Gam cytidine base editor system without GFP for sorting of transfected cells^35^ was very variable and overall low (0.6–13%). Improved versions of the cytosine base editors, such as BE4max with a co-expressed GFP marker^36^, will likely improve editing efficiencies. We also experienced unintended editing of bases at a close distance to the target base in two instances. This underlines the importance of, (if possible), choosing a target base in a genomic area with the least number of possible unintended targets. There are now many improved versions of the cytosine base editor available, offering high editing efficiency and reduced off-target effects and bystander editing. It is important to carefully select the most appropriate base editor^37^.

In line with previous reports of disturbed retrograde IFT in fibroblasts from human SRTD cases^9,38^ and knockout mouse models^17^ we observed disturbed retrograde IFT in all three dynein-2, intermediate chain mutants. WDR60p.Ala911Val and WDR34p.Arg183Trp mutant cells exhibited a small but statistically significant reduction in cilia length compared to control cells; no difference regarding the ciliation rate was observed for any mutant clone compared to controls. In line with this finding, analyses of fibroblasts from patients with WDR34 mutations displayed shorter cilia^7^ and fibroblasts from SRPS patients with WDR60 mutations showed ciliogenesis defect with few cells forming stumpy cilia, while WDR60 mutant JATD fibroblasts were only mildly affected^9^. This suggests a connection between the extent of dynein-2 loss of function and cilia defects, further supported by the finding that ciliogenesis is not affected in *DYNC2H1* mutant fibroblasts carrying hypomorphic mutations^5^ while *Dync2h1* mouse mutant fibroblasts with complete Dync2h1 loss of function only form ciliary stumps^39^. Interestingly, however, knockout of *WDR34* in human telomerase-immortalized RPE1 (hTERT-RPE1) cells has been reported to affect the axoneme extension, with cells showing only pre-ciliary vesicles, while *WDR60* knockout in the same cells did not affect cilia formation and only had a mild impact on cilia length^18^. The underlying reason for this difference has remained unexplained. We also detected impaired reduction of GLI3 repressor to full-length ratio upon addition of the hedgehog agonist SAG, as well as increased GLI3 full-length amounts in all three mutants in comparison to control cells, recapitulating the hedgehog signaling defect observed in dynein-2 intermediate chain mutant patient cells^9^, knockout cells^18,40^, and animal models^17^.

Transcriptome analyses did not reveal any influence of the mutations on the expression level of dynein-2 complex or IFT genes. However, expression of *DCDC2A*, a hepato-renal ciliopathy gene, was significantly lower in all mutants compared to controls. DCDC2A co-localizes with acetylated tubulin along the ciliary axoneme as well as spindle microtubules and is thought to play a role in microtubule polymerization^41^. DCDC2A also interacts with DVL1-3, suggesting a role in Wnt signaling regulation^29^. In line with this, Schueler et al. suggest that DCDC2A dysfunction results in a ciliogenesis defect in kidney cells with constitutive activation of the canonical Wnt signaling pathway^29^. While patients harboring biallelic *DCDC2* mutations do not usually exhibit a skeletal phenotype, nephronophthisis (like) or cystic renal disease, as well as rarely cystic-fibrotic hepatic changes, can be observed in a subset of SRTD patients, especially in fetuses with an overall severe phenotype. Potentially, reduced DCDC2 expression could contribute to renal and hepatic findings in SRTD. Consistent with this, the patient carries WDR60p.Ala968Val homozygously presented with chronic hepatic disease (Supplementary information patient phenotype file, Table 1). Human patients carrying WDR34 p.Gly393Ser homozygously were fetuses^8,14^, and the patient with the homozygous WDR34 p.Arg182Trp variant passed away at 4 months of age^42^ without any reported overt liver or kidney phenotype; however, the development of kidney or liver anomalies at a later age could not be ascertained.

We also observed consistent downregulation of *Rab6b* in all mutant clones versus controls in both ciliated and non-ciliated cells. RAB6B is a small GTPase important for Golgi to ER transport^43^, mediated by COPI vesicles^44^. RAB6B interacts with Bicaudal D-1, a binding partner of the dynein-1/dynactin complex^30^ as well as with Roadblock-1 (DYNLRB1)^45^, a light chain component of both cytoplasmic dynein-1 and dynein-2 (IFT-dynein). RAB6B- Bicaudal-D1 complex is proven to co-localize at the Golgi^30^. In dynein-2, DYNLRB1 interacts with the N-terminal domains of WDR34 and WDR60, and these interactions enable the conformational change of dynein-2 motor during IFT transport, as well as cargo binding^46,47^. RAB6B shows 91% similarity with RAB6A, and both interact with Rabbkinesin-6^43^, with no specific RAB6B antibody available. Unfortunately, this prevented us from evaluating protein levels and subcellular localization of RAB6B in mutant clones versus controls. Our results suggest impaired Golgi recovery after stress in mutant cells. In line with impaired Golgi protein transport functions under stress, we observed reduced expression of several other Golgi-associated proteins, including ELMOD1, in all dynein-2 mutants (both ciliated and non-ciliated cells) compared to controls. The ELMOD1 is a GTPase-activating protein that acts mainly on ADP-ribosylation factor (ARF) family GTPases, and knockout affects primary cilia formation, reducing ciliary ARL13B expression and resulting in accumulation of INPP5E and IFT140 at the Golgi^31^. Knockout mice present with fused and elongated stereocilia in the inner ear and disturbed intracellular vesicle trafficking in knockout cells have been described^48^. Further, we detected up-regulation of RGS2, a regulator of G protein signaling and inhibitor of COPI vesicles binding to Golgi in all mutants^23^.

Due to the extensive amount of extracellular matrix proteins required for cartilage and bone formation and maintenance, proper function of protein transport and posttranslational modification at the Golgi is essential for skeletal development. It comes as no surprise that defective protein processing and transport, as well as Golgi dysfunction, have been observed with many skeletal dysplasias/developmental skeletal conditions, including geroderma osteodysplasticum (OMIM: 231070), caused by dysfunction of GORAB, a trans-Golgi and centrosome RAB6-interacting protein^49^ and *ARCN1-*associated syndromic rhizomelic short statur*e*^50^. *ARCN1* encodes delta-COP, localizing at the Golgi, ER, and intracellular vesicles, and plays a role in ER stress regulation and ER-Golgi protein transport.

Previous studies have further suggested links between ciliary proteins, Golgi function, and skeletal dysplasias. Grissom et al. have suggested that the dynein-2 light intermediate chain DYNC2LI1 co-localizes with DYNC2H1 at the Golgi and mediates cargo-loading to the dynein-2 complex at the Golgi^51^. Follit et al. demonstrated that TRIP11 (GMAP-210), a Golgi-associated microtubule-binding protein, anchors IFT20 to the Golgi^52^. TRIP11 is able to recruit gamma-tubulin-containing protein complexes to the Golgi membrane independent of microtubule integrity, and TRIP11 depletion causes Golgi fragmentation^53^. Dysfunction of TRIP11 causes a severe chondrodysplasia phenotype presenting with thoracic dysplasia resulting in lung hypoplasia in humans and mice^54,55^ with cells showing a defective Golgi architecture, impaired Golgi-mediated glycosylation, and intracellular accumulation of the extracellular matrix protein perlecan^55^.

In line with a mildly perturbed COPI-Golgi function in our mutants, we found reduced COPI-Golgi recovery abilities after monensin stress application in mutants versus controls, which could be partially rescued by expression of wild-type WDR34 or WDR60. Overall, WDR34 Gly394Ser mutant cells displayed a more severe phenotype compared to the other two mutants, while described human phenotypes for the two different WDR34 alleles do not seem to differ with regard to the severity of death in utero or within the first months of life^8,14^. We cannot exclude that this could be attributed to the additional effects of the unintentional heterozygous WDR34 Gly393Asp change in our clone. While GO term analyses of mutant versus wildtype clones revealed down-regulation of pathways essential for angiogenesis/vasculature development as well as cell and tissue migration in both ciliated and non-ciliated cells, we observed lower expression of *Vegfa* only in mutant ciliated cells compared to ciliated controls. While *Vegfa* knockout is embryonically lethal in mice, hypomorphic loss of function results in angiogenesis defects, including cartilage vascularization defects^56^. Reduced *Vegfa* expression has been further found at the growth plates of *Kif3a* knockout mice. *Kif3a* encodes for a subunit of the anterograde IFT-motor kinesin, and *Kif3a* loss-of-function phenotypes in mice resemble dynein-2 dysfunction phenotypes, including hedgehog signaling defects, growth plate dysfunction, and skeletal defects^57^. This could indicate a role of cilia or IFT in regulating Vegfa expression.

Limitations of this study consist of the presence of the additional unintended heterozygous missense change *Wdr34* c.1178 G > A in the *Wdr34* c.1180 G > A, p.Gly394Se clones, as well as representing in vitro data, which ideally should be confirmed in the future in vivo using mouse models carrying human disease alleles. Further, as some cell signaling, such as hedgehog signaling, is not intrinsically activated in vitro and requires stimulation, cell signaling changes in mutant cells can be missed using transcriptome analysis in non-stimulated cells. Accordingly, we did not detect changes in hedgehog signaling in RNA sequencing experiments, while we observed hedgehog defects after stimulation using GLI3 western blot analysis (Fig. 3H, I). Possibly, there are other cell signaling defects we did not detect due to a lack of stimulation/activation of those pathways. This will require future pathway-specific analyses.

In summary, in this study, we successfully created SRTD disease models carrying human missense alleles using base editing. We confirmed retrograde IFT defects as well as disturbed hedgehog signaling and further detected evidence for COPI–Golgi perturbation associated with human disease alleles, which could, combined with changes in the cytoskeleton and extracellular matrix composition, contribute towards the severe skeletal phenotype associated with dynein-2 intermediate chain dysfunction.

## Materials and methods

### Ethics statement

The methods were performed in accordance with relevant guidelines and regulations, and genetic diagnostics were performed under the Diagnostic Innovation Program Radboudumc Nijmegen (Ethics committee Arnhem-Nijmegen).

### Human DNA samples

Written consent was obtained from all participants or their legal guardians. A genetic diagnostic was performed at Radboudumc in Nijmegen under the Diagnostic Innovation Program Radboudumc Nijmegen. Genomic DNA extraction was performed with the Qiagen genomic DNA extraction kit (Qiagen, Germantown, MD, USA). DNA concentrations were determined by Nanodrop (Thermo Fischer Scientific, Waltham, MA, USA).

### Whole exome sequencing

Exome sequencing and data analysis were performed as previously described^58–60^. In brief, 2-5 micrograms of DNA from the index case were used for whole exome sequencing (WES) at Novogene, Hong Kong. Agilent SureSelect Human All Exon V5 Kit (Agilent, Santa Clara, California, USA) was used for capture and sequencing performed on an Illumina HiSeq 2500 machine (Illumina, San Diego, California, USA). Paired-end sequencing resulted in sequences of 150 bases from each end of the fragments. UCSC hg19 was used as a reference genome. VarScan version 2.2.5 and MuTec and GATK Somatic Indel Detector were used to detect SNVs and InDels. Variants were then filtered in-house to select variants with a minor allele frequency (MAF) of <1% in public control databases, including dbSNP, ExAc, and gnomAD (https://www.ncbi.nlm.nih.gov/snp/; https://gnomad.broadinstitute.org/). Remaining variants were first filtered for known disease-causing genes with an emphasis on diseases compatible with the patient phenotype (SRTD). Homozygous variants were prioritized due to the consanguinity of the family. Additionally, visual BAM file inspection was performed for homozygous CNVs in genes previously associated with SRTD, and exome data files were further analyzed for the presence of heterozygous CNVs using Exome Depth^61^.

### Sanger sequencing

Primers specific to the locus of the novel WDR60 c. 2903 C > T, p.Ala968Val variant was designed using primer-BLAST (https://www.ncbi.nlm.nih.gov/tools/primer-blast/) and PCR amplification of the region was performed with Q5 DNA polymerase (New England Biolabs, Ipswich, Massachusetts, USA). The PCR product was then sequenced by reverse primer, using Sanger´s chain termination method. (Primer sequences Forward- 5′CTCTGGCTGCTGAGATCAAGT3′; 5′AAAGACCGTAAGTGGCACCC3′).

### CRISPR–Cas9 mediated mutation creation in Cilia–APEX–IMCD3

Cilia–APEX–IMCD3 (Inner Medullary Collecting Duct) cells were a kind gift from Maxence Nachury, Stanford University School of Medicine, USA. First, we determined the corresponding positions of the human disease alleles in the mouse genome (mutation taster program) (Fig. 2D–F). Missense changes created in Cilia–APEX–IMCD3 cell lines are labeled throughout the manuscript with reference to the mouse genome. Protospacer sequences and corresponding oligos used to generate spacer sequences are provided in the Supplementary Table 2. The selected target sequences (protospacer along with the PAM sequence) were checked for specificity against the mouse genome (GRCm39) using NCBI BLAST. The gRNA binding regions were checked for the presence of single-nucleotide polymorphism (SNP) by PCR (One Taq 2× master mix (New England Biolabs, Ipswich, Massachusetts, USA) and subsequent Sanger sequencing of the PCR product (primers designed by Primer-BLAST, sequences are available on request). The oligos, along with the overhang sequence, were synthesized by Eurofins Genomics (Eurofins Genomics, Ebersberg, Germany). The oligos were annealed and ligated into the gRNA expression vector, BPK1520 (Plasmid #65777, Addgene, Watertown, Massachusetts, USA), using the Anza T4 DNA ligase master mix (Thermo Fisher Scientific, Waltham, Massachusetts, USA) after digesting the vector with BsmBI restriction enzyme (New England Biolabs, Ipswich, Massachusetts, USA).

Cilia–APEX–IMCD3 cells were transfected with 4 µg of the cytosine base editor encoding plasmid BE4-Gam (kind gift from David Liu; Addgene Plasmid #100806, Addgene, Watertown, Massachusetts, USA) and 1 µg of BPK1520 with respective target oligo inserts using jetPRIME transfection reagent (Polyplus-transfection, Illkirch-Graffenstaden, France) following the manufacturer’s instructions. Forty-eight hours after the first transfection, cells were split and plated at around 40% confluency and re-transfected the following day. Single cell sorting was performed 72 h after the second transfection (BD FACSAria III Cell Sorter, BD Biosciences, Franklin Lakes, New Jersey, U.S.A) and cells were collected in fifteen 96-well plates. For controls, cells were transfected with plasmid BE4-Gam and 1 µg of BPK1520 without any specific target sequence oligos. Single clones were picked approximately 3 weeks later, and part of the cells were used for genotyping using Sanger sequencing (primer sequences are given in Supplementary Table 2) (Supplementary Fig. 1).

### Immunofluorescence analysis

Ciliation was induced by 32 h of serum starvation. Cells were then washed with PBS and fixed with ice cold 100%methanol. Fixed cells were stained with rabbit polyclonal IFT88 (1:100 dilution; 13967-1-AP, Proteintech, Rosemont, IL, USA) and mouse monoclonal gamma tubulin primary antibodies (1:200 dilution; T6557, Sigma, Taufkirchen, Germany), overnight at 4 °C after permeabilizing with 0.5% Triton-X 100. Cells were then washed three times with 1× PBS and incubated with Alexa Fluor 568 goat anti-rabbit (1:500; A11011, Life Technologies, Carlsbad, CA, USA) and Alexa Fluor 633 goat anti-mouse IgG1 (1:500; A21126, Life Technologies, Carlsbad, CA, USA) secondary antibodies for 1 h. Cells were finally washed three times with 1× PBS and mounted in Vecta shield with DAPI (Vector Laboratories, Burlingame, USA). Images were taken using a Zeiss LSM NLO inverted microscope (Carl Zeiss Microscopy, Jena, Germany) with a 63× objective. Image analysis was performed using ImageJ software. For investigations of putative retrograde IFT defects, the accumulation of IFT88 at the ciliary tip in 130 cilia per mutant clone and control clone was analyzed. For cilia length analyses, the length of the 100 cilia per clone was measured using Image J, and for ciliation efficiency, 100 cells were checked for cilia formation.

### Western blot

Control and mutant cells were serum-starved in a medium containing 0.2% FBS for 24 h. For GLI3 western blots, cells were then treated with 500 nm SAG. After 24 h, cells were lysed in RIPA lysis buffer (Abcam, Cambridge, UK), followed by total protein concentration estimation (Bradford assay). 30 µg of total protein was then separated in NuPAGE Bis–Tris gel (4–12%, Invitrogen, Waltham, Massachusetts, USA), followed by western analysis using goat polyclonal GLI3 antibody (1:1000 dilution; AF3690, R&D Systems, Minneapolis, Canada). The intensity of bands was then quantified by Image J (densitometry). For PVRL1 western blot analysis, a mouse monoclonal antibody was used (1:1000; MABT61, Sigma, Taufkirchen, Germany), and proteins were separated in Tris-Glycine gel 4–20% (Invitrogen, Waltham, Massachusetts, USA). Rabbit polyclonal beta-actin antibody (1:10000; Ab8227, Abcam, Cambridge, UK) was used as a loading control.

### RNA sequencing

For RNA sequencing, RNA was extracted from cells grown in 6-well plates using the RNeasy Mini Kit (Qiagen, Germantown, MD, USA) following the manufacturer’s instructions. To induce ciliogenesis, the cells were cultured in starvation medium (0.2% FCS, instead of 10%) for 50 h. To control for proper ciliation of ciliated samples, a coverslip was placed on one well of the 6-well plate. The cells were then fixed in 4% PFA and stained with rabbit polyclonal ARL13B primary antibody (1:100; 17711-1-AP, Proteintech, Rosemont, Illinois, USA) and checked for ciliation before RNA extraction. The quality and quantity of total RNA were measured using the High Sensitivity RNA Screen Tape Analysis (Part number 5067-5579, Agilent Technologies, Santa Clara, California, United States) and Qubit RNA HS Assay Kit (Thermo Fisher Scientific, Waltham, MA, USA, Q32852), respectively. RNA preparation and RNA sequencing were performed as previously described^62^. RNA sequencing of four different control clones and three different mutant clones with four technical replications each was performed at Novogene, Hong Kong, on a NovaSeq PE150 sequencer (12 G raw data per sample).

### Transcriptomics bioinformatics

RNA sequence analyses were performed in-house as previously described^63^. In brief, sequence reads were mapped to the mouse reference genome GRCm38/mm10 (iGenomes, Illumina; chromosomes 1–19, X, Y, M) using the Rsubread v1.28.1 package in R v3.4.4. Gene counts were retrieved from the mapping, applying the Rsubread::featureCounts function with the iGenomes reference genome gtf file for annotation (archive-2015-07-17-32-40 and archive-2015-07-17-33-26 for GRCM38). For differential expression analysis, DESeq2 v1.18.1 was used. First, the counts were normalized by library size (DESeq2::estimateSizeFactors) and by gene-wise dispersion (gene-wise geometric mean over the samples; DESeq2::estimateDispersions), then the differential expression analyses were performed applying negative binomial generalized linear model fitting and Wald statistics on the normalized count data (DESeq2::DESeq2). Subsequently, the results were filtered for adjusted *p* < 0.05, and the log2 fold change values (log2FC) are indicated in the figure legends. Interactive volcano and MDS plots were generated using the R package Glimma (1.10.1) and heatmaps using pheatmap (v1.0.12). For GO term enrichment analyses, gene symbols were converted to gene IDs using biomaRt (v2.38.0) and subsequently, enrichment was assessed and visualized using GO.db (3.7.0) and clusterProfiler (3.10.1).

### Golgi stress assay and rescue

Cells were treated with 10 µM monensin for 16 h, followed by washing twice with 1XPBS and incubating the cells in fresh medium for 6 h. After 6 h the cells were washed in 1× PBS and fixed in 4% PFA followed by staining with rabbit polyclonal beta COP antibody (1:100; Ab2899, Abcam, Cambridge, UK) after permeabilizing with 0.5% Triton-X 100. Alexa Fluor 488 goat anti-rabbit (1:500; A11008, Life Technologies, Carlsbad, California, USA) was used as the secondary antibody. Cells were imaged and analyzed as explained above. A total of 25 cells per condition was counted in one experiment to calculate the dispersed COPI compartment.

For the rescue experiment, mutant cells were first transfected with the respective wild-type plasmid using jetPRIME transfection reagent (Polyplus-transfection, Illkirch-Graffenstaden, France) according to the manufacturer’s instructions for WDR34p.Arg183Trp and WDR34p.Gly394Ser mutants, cells were transfected with 1 µg of WDR34 human Myc-DDK-tagged ORF Clone (RC204288, OriGene, Rockville, Maryland, USA) and for WDR60p.Ala911Val mutant cells were transfected with 1 µg of WDR60 Mouse Myc-DDK-tagged ORF Clone (MR217536, Origene, Maryland, USA). After 24 h, cells were treated with 10 µM monensin, and immunofluorescence analysis was done as explained above. To visualize transfected cells in rescue experiments, mouse monoclonal FLAG primary antibody (1:100; F1804-50UG, Sigma, Taufkirchen, Germany) and Alexa Fluor 568 Goat anti-mouse secondary antibody (1:500; A11031, Life Technologies, Carlsbad, California, USA) were used along with COP antibody and corresponding secondary antibody. Only cells that indicated positive flag staining were analyzed for the effect of the wild-type plasmid on the COPI compartment.

### Statistical analysis

For the analysis of IFT88 accumulation at the tip of the cilia, Fisher’s exact test was performed, and 130 cells were analyzed per genotype. Student’s *t*-test was performed for the cilia length analysis (100 cells per genotype analyzed). Significance of ciliation efficiency in mutants versus control was determined by performing Fisher’s exact test (100 cells per genotype analyzed). For the analysis of GLI3 repressor to GLI3 full-length ratio in Fig. 3I, two-way annova with Bonferroni’s multiple comparisons test was performed. For the analysis of the percentage of cells with dispersed COPI compartments after the monensin stress assay in mutants versus controls in Fig. 5I, one-way ANOVA with Dunnett’s multiple comparisons test (25 cells per genotype analyzed) was performed. For the rescue experiment in Fig. 5M, Fisher’s exact test was performed after calculating the percentage of cells with dispersed COPI compartments (25 cells per genotype analyzed). Raw data used for the figures are provided in the Supplementary Data 7 and 8.

### Web resources

https://www.ncbi.nlm.nih.gov/snp/

https://gnomad.broadinstitute.org/

https://blast.ncbi.nlm.nih.gov/Blast.cgi

https://www.mutationtaster.org/

https://www.ncbi.nlm.nih.gov/tools/primer-blast/

http://genetics.bwh.harvard.edu/pph2/

### Reporting summary

Further information on research design is available in the Nature Portfolio Reporting Summary linked to this article.

## Supplementary information

Supplemental information

Description of Additional Supplementary Files

Supplementary data 1

Supplementary data 2

Supplementary data 3

Supplementary data 4

Supplementary data 5

Supplementary data 6

Supplementary data 7

Supplementary data 8

Reporting Summary

## Data Availability

The datasets generated during and/or analyzed during the current study are available in the GEO database, with the GEO accession number GSE208712.
