# Supplementary material for: Base editing-derived models of human WDR34 and WDR60 disease alleles replicate retrograde intraflagellar transport (IFT) and hedgehog signaling defects
Source: Commun Biol. 2026 Jul 1;9:889. doi: 10.1038/s42003-026-10507-2 (PMC13328300; doi:10.1038/s42003-026-10507-2)
Supplement: Supplementary file 1 — Supplemental information [file 42003_2026_10507_MOESM1_ESM.pdf]

Supplementary Information

List of Supplementary Data files

Supplementary Data 1	WDR60p.Ala911Val_ciliated cells GO_BP
Supplementary Data 2	WDR34p.Arg183trp_ciliated cells GO BP
Supplementary Data 3	WDR34p.Gly394Ser_ciliated cells GO_BP
Supplementary Data 4	Diffrentially expressed genes WDR60p.Ala911Val_ciliated cells
Supplementary Data 5	Diffrentially expressed genes WDR34p.Arg183trp_ciliated cells
Supplementary Data 6	Diffrentially expressed genes WDR34p.Gly394Ser_ciliated cells
Supplementary Data 7	Raw data used forcreating the Figures
Supplementary Data 8	Raw data for Figure 4 F

A detailed description of the clinical phenotype of SI_36

The index was the second child of a consanguineous couple (second-degree cousins). His mother previously had given birth to a healthy boy and reported one miscarriage where no genetic testing was performed. The mother was referred to our clinic for genetic counseling when she was 26week pregnant for the index. On detailed prenatal ultrasonography, the fetus presented with short and mildly bowed extremities (femur, humerus tibia) and mild angulation on the humerus, normal head circumference, frontal bossing, mildly narrow thorax (increased cardiothoracic index: 66%) and right pes equinovarus. Chromosome analysis and FGFR3 gene analysis from cord blood were normal. He was born at term with a birth weight of 3400 gr, birth length: 51 cm, birth OCD: 38 cm and stayed at the NICU for 2 weeks because of respiratory distress and direct hyperbilirubinemia (cholestasis). He presented with a narrow thoracic cage, predominantly rhizomelic short extremities short hands and feet and brachydactyly, partial cutaneous syndactyly of 2nd and 3rd toes, bilateral palmar simian creases, distally placed small nails, especially toenails were deeply placed and hypoplastic, high palate with normal frenula, mild umbilical hernia and normal genitalia. Measurements at 17 days of age were Wt: 3260 gr, ht: 51.5 cm, OFD: 35.5 cm. He was clinically diagnosed with Short Rib Thoracic Dysplasia /Jeune Syndrome.

Newborn Echocardiography showed mild peripheral pulmonary stenosis, a control at 1 year of age showed no abnormalities. Newborn abdominal ultrasound showed mild hepatomegaly, hydronephrosis of the left kidney but no bile duct abnormality. A control showed normalization of the left sided hydronephrosis. Ophthalmological examination and brain ultrasound revealed no abnormalities.

During follow-up, he developed several lower airway infections and he suffered from chronic respiratory distress. Sternotomy and thoracic expansion surgery were performed at the age of 7 months. He was last evaluated when he was 14 months old, measurements were height: 69-70 cm (< 3. Centile), weight: 6730 gr (< 3rd centile), OFD: 43.5 cm when he still experienced respiratory distress, mild cyanosis, narrow thoracic cage with distended abdomen. His nasal base appeared flat, had frontal bossing, hypertelorism, and defective wedged enamels of his teeth. He was admitted several times to the ICU for respiratory insufficiency. A thoracic CT scan at the age of 6 months revealed a narrow thoracic cage especially on superior and anterior parts, relative cardiomegaly, right aberrant subclavian artery, ground-glass opacifications in some segments of both lungs and linear opacities showing linear subpleural atelectasis.

He further suffered from chronic hepatic disease and portal hypertension and he had paracentesis twice for ascites. Portal vein Doppler and ultrasound at the age of 9 months revealed hepatosplenomegaly, parenchymal heterogeneity of the liver, decreased caliber of the portal vein, increased echogenity of periportal regions. Decreased blood flow through the portal vein and ascites.

He was trying to stand up and walk at 14 months of age. His voice was low pitched due to longterm intubation. At 1.5 years of age, he sadly passed away.

Three years later the mother was pregnant again with twins, however, both fetuses died in utero around five months into the pregnancy. One fetus was macerated at birth. The other fetus showed mildly narrow thorax, short extremities and brachydactyly of the hands. No autopsy was performed**.**

**Description**	**geneID**
adhesion of symbiont to host	Ace2/Gbp7/Gbp3/Nectin1/Gbp2/Gbp6
response to interferon-beta	Ifi203/Tgtp1/Tgtp2/Gbp3/Gbp2/Gbp6/Ifi47/Ifitm3
response to interferon-gamma	Tgtp1/Gbp7/Gbp3/Gbp10/Gbp2/Gbp6/Snca/Ifitm3/Gbp4
defense response to protozoan	Gbp7/Gbp3/Gbp10/Gbp2/Gbp6
muscle cell proliferation	Il15/Ace2/Angpt1/Esr1/Tenm4/Retn/Tgm2/Gper1/Fgf1
organ growth	Col6a2/Ddr2/Col6a1/Esr1/Tenm4/Rgs2/Matn2/Fgf1
axon development	Dclk1/Tspan2/Epha7/Map2/Nectin1/Gdnf/Ispd/Flrt2/Adcy1/Ust/Matn2/Bex1
urogenital system development	Angpt1/Kif26b/Epha7/Nfia/Gdnf/Esr1/Serpinb5/Hey1/Irx1/Fgf1

regulation of synapse assembly	Ptprd/Epha7/Adgrl3/Nectin1/Flrt2/Snca
extracellular structure organization	Lox/Abca1/Col11a1/Ddr2/Eln/Abi3bp/Serpinb5/Flrt2/Col16a1
chondrocyte morphogenesis involved in endochondral bone morphogenesis	Col6a2/Col6a1/Matn2
regulation of monooxygenase activity	Gdnf/Esr1/Atp2b4/Snca
nephron tubule morphogenesis	Kif26b/Gdnf/Hey1/Irx1/Fgf1
regulation of G protein-coupled receptor signaling pathway	Rgs9/Kctd12b/Rgs2/Atp2b4/Snca/Gper1
growth plate cartilage morphogenesis	Col6a2/Col6a1/Matn2
endochondral bone growth	Col6a2/Ddr2/Col6a1/Matn2
glycosaminoglycan metabolic process	Dse/Il15/Hexa/Dcn/Angpt1
chondrocyte development	Col11a1/Col6a2/Col6a1/Matn2
regulation of protein autophosphorylation	Gpnmb/Vegfc/Syk/Epha7

Supplementary Table1. Gene ontology term biological process; upregulated in ciliated cells

**Protospacer and PAM sequences**	**Primer Sequences**
**WDR60 p.Ala911Val**<br>CCTGCAGTGTTCCTGGTCCAGG<br><br>5’CACCCCTGCAGTGTTCCTGGTCC 3’<br>5’AAACGGACCAGGAACACTGCAGG3’	Forward- 5’TATGTTCAGAAAACAAGAGCAAGCC 3’<br>Reverse- 5’AATTTAAGAAGAGCAAGGATGAGGG 3’
**WDR34 p.Arg183Trp**<br>TGGTCGGTGAGTGAGAGCTGG<br><br>5’ CACCTGGTCGGTGAGTGAGAGC 3’<br>5’ AAACGCTCTCACTCACCGACCA 3’	Forward- 5’GTCTTAGGTGCCCGGTGAAA 3’<br><br>Revere - 5’ TGGTGAGTGGAGACCACAGA 3’

**WDR34 p.Gly394Ser**<br>CCTTCTCTCCCCATGGTGGCCC<br><br>5´CACCGGGCCACCATGGGGAGAGA3´<br>5´AAACTCTCTCCCCATGGTGGCCC 3´	Forward- 5’ GCTTCCCCCTCAAGTGTTCC 3’<br><br>Reverse- 5’ TGTATGGCTTGTGCCTACCTTC 3’

Supplementary Table 2. Protospacer and PAM sequences (blue) of the genomic loci used for CRISPR single base editing with target base shown in red. The corresponding oligos used to generate the spacer sequences are given below (overhang sequences in green). Primer sequences used to amplify the genomic loci are also given.

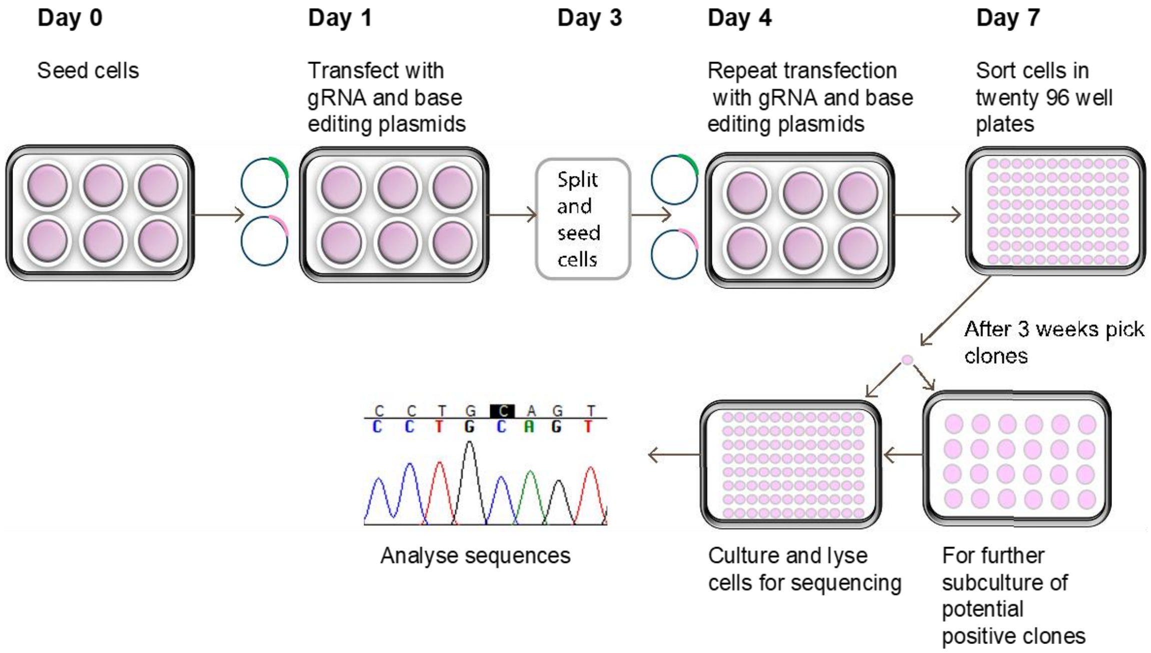

Supplementary Fig. 1 CRIPSR single base editing workflow. Cells were transfected twice with cytosine base editor and gRNA encoding expression plasmids. Single cells were FACS sorted 72 hours after the second transfection round and allowed to grow until confluency, followed by clone picking and Sanger sequence analysis to identify edited clones. This figure was created using PowerPoint and Adobe Illustrator. The chromatogram is a screenshot of the Sanger sequence analysis from our experiment. AI tools were not used to generate this figure.

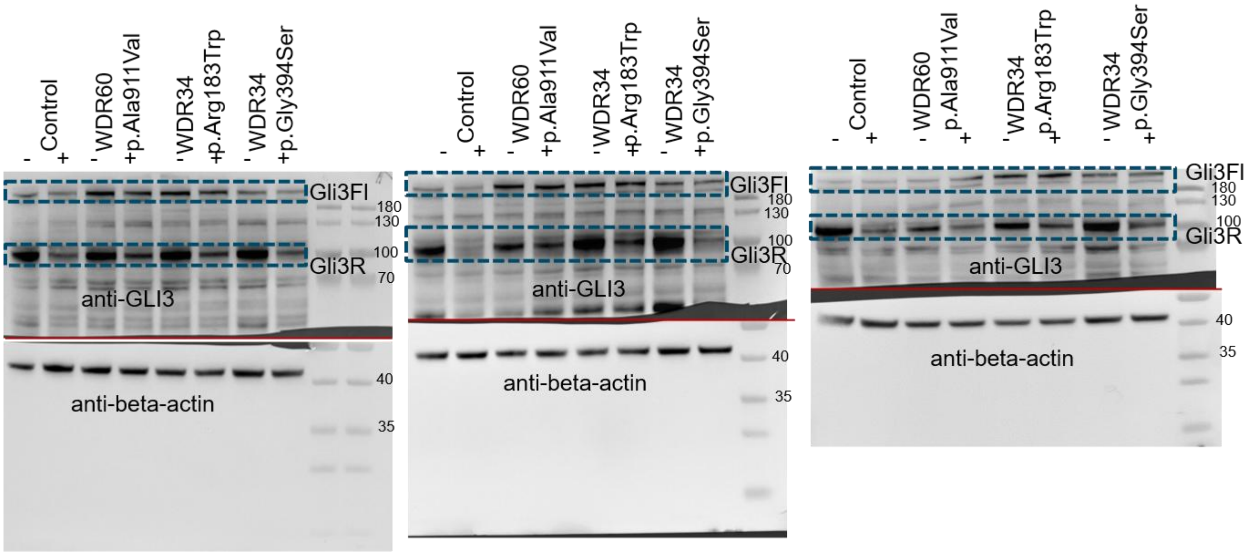

Supplementary Fig.2. Full gel images showing expression levels of GLI3 full length. GLI3 repressor and beta-actin as a loading control. GLI3FL: GLI3 full length; GLI3R: GLI3 repressor. n=3 independent experiments. beta-actin was assessed by cutting the blot after transfer (red line indicates the cut) This blot was cut after the transfer, due to the incompatibility of the antibodies namely, goat anti-gli3 and secondary Ab anti rabbit HRP which was used to detect beta actin.

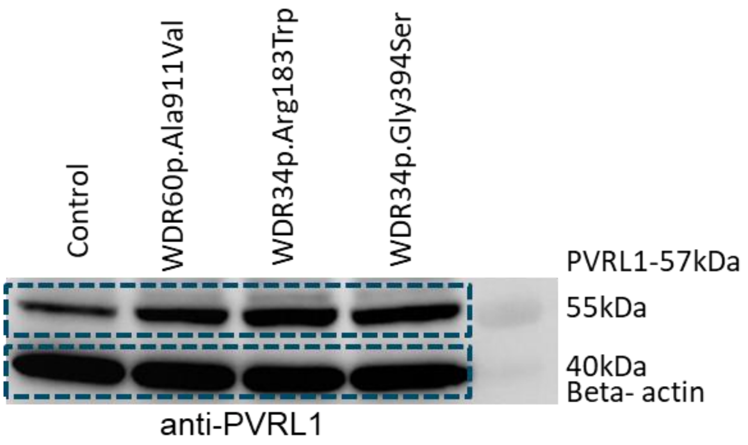

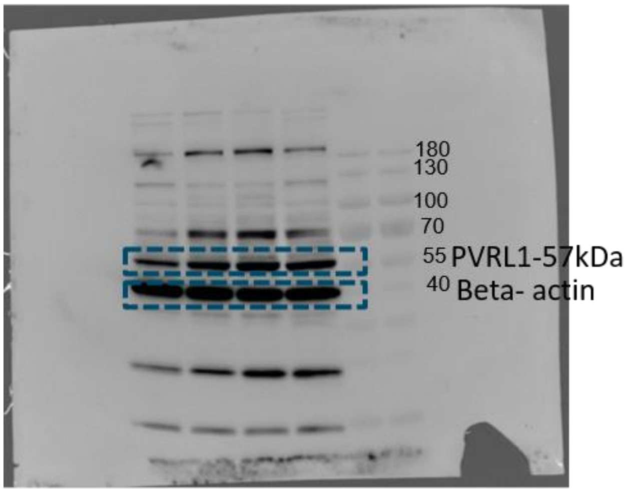

Supplementary Fig. 3. Full gel images of PVRL1 western blot. Beta-actin was used as the loading control.

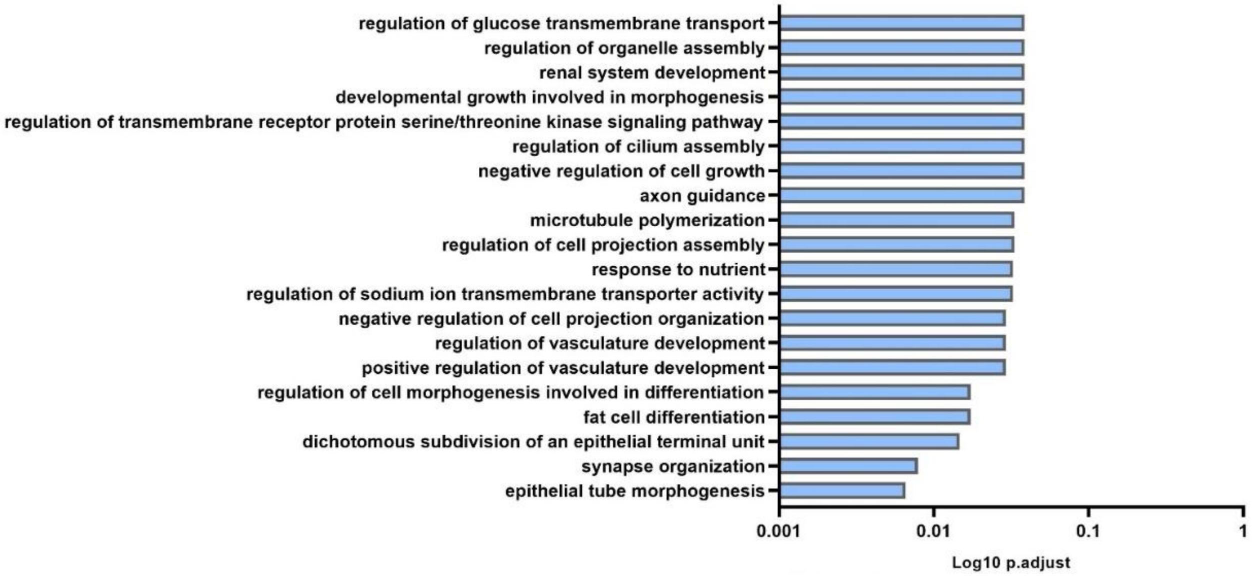

Supplementary Fig. 4. GO term biological process differentially regulated in WDR60 p.Ala911Val clone in ciliated cells (The top 20 Gene Ontology (GO) terms are shown after similar terms have been removed. The full list of GO terms is provided in Supplementary Data 1)

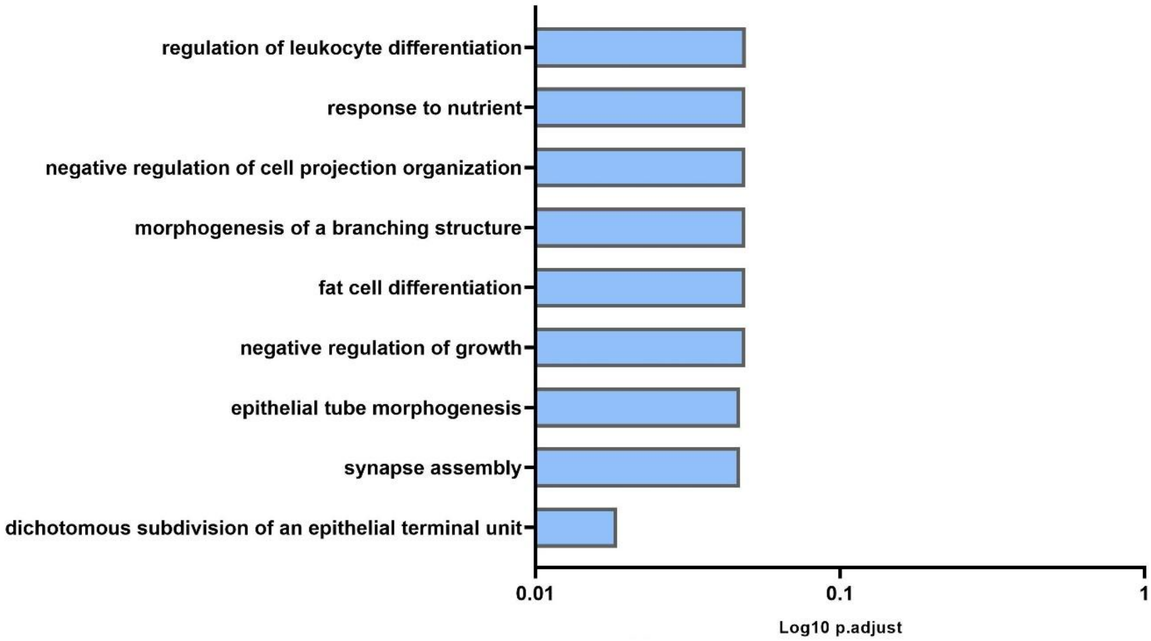

Supplementary Fig.5. GO term biological process down regulated in WDR60 p.Ala911Val clone in ciliated cells.

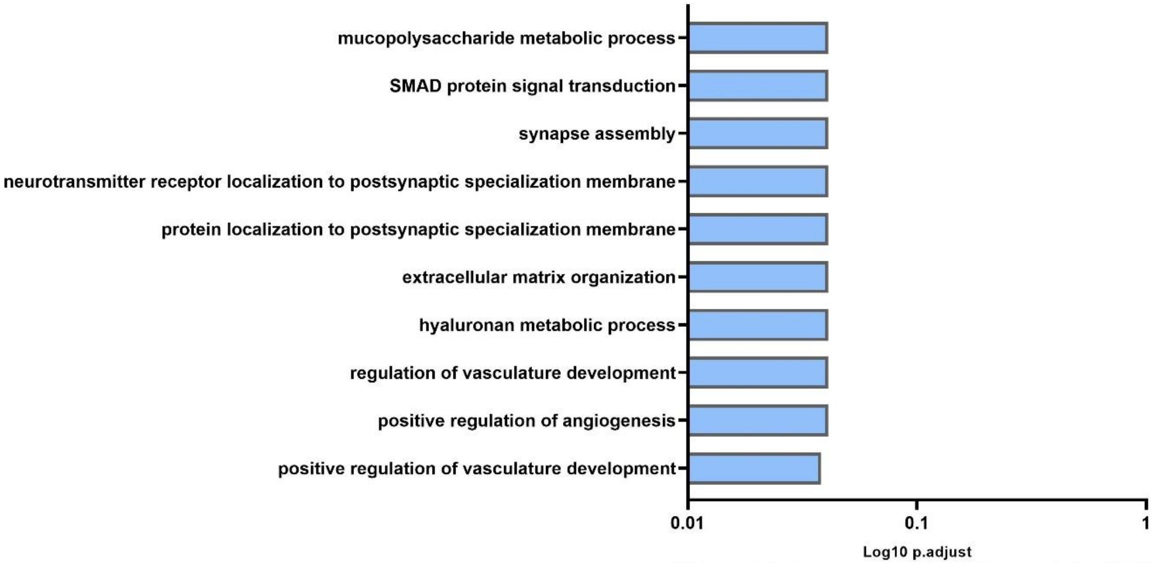

Supplementary Fig.6. GO term biological process differentially regulated in WDR34 p.Arg183Trp clone in ciliated cells. The data used to generate the graph is provided in the Supplementary Data 2.

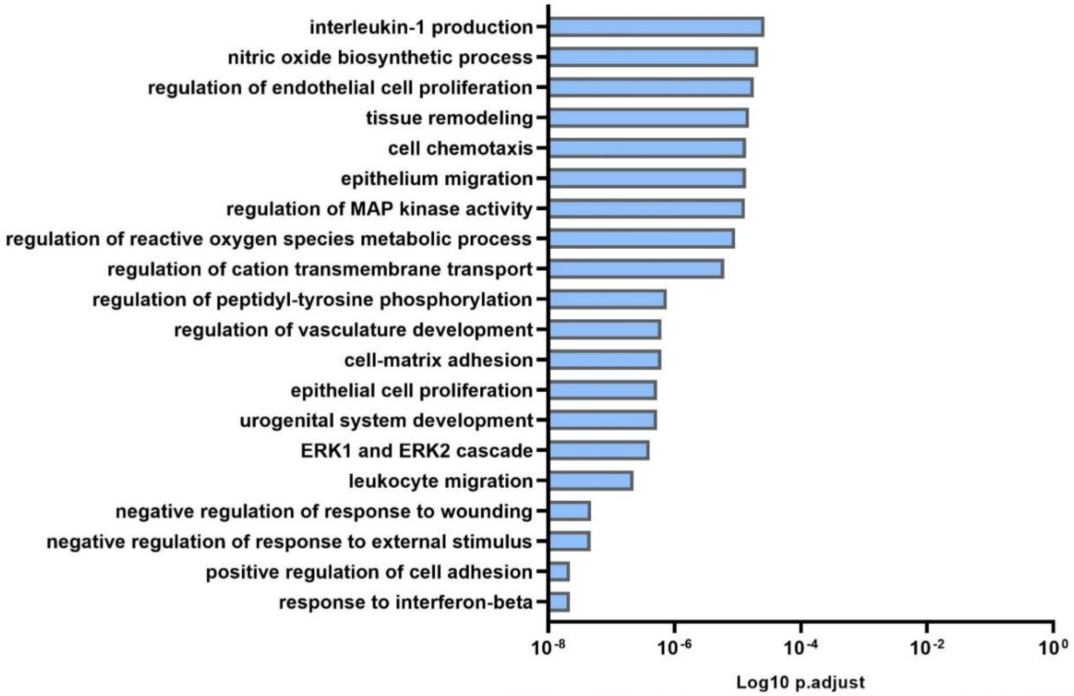

Supplementary Fig. 7. GO term biological process differentially regulated in WDR34 p.Gly394Ser clone in ciliated cells (The top 20 Gene Ontology (GO) terms are shown after similar terms have been removed. The full list of GO terms is provided in Supplementary Data 3).

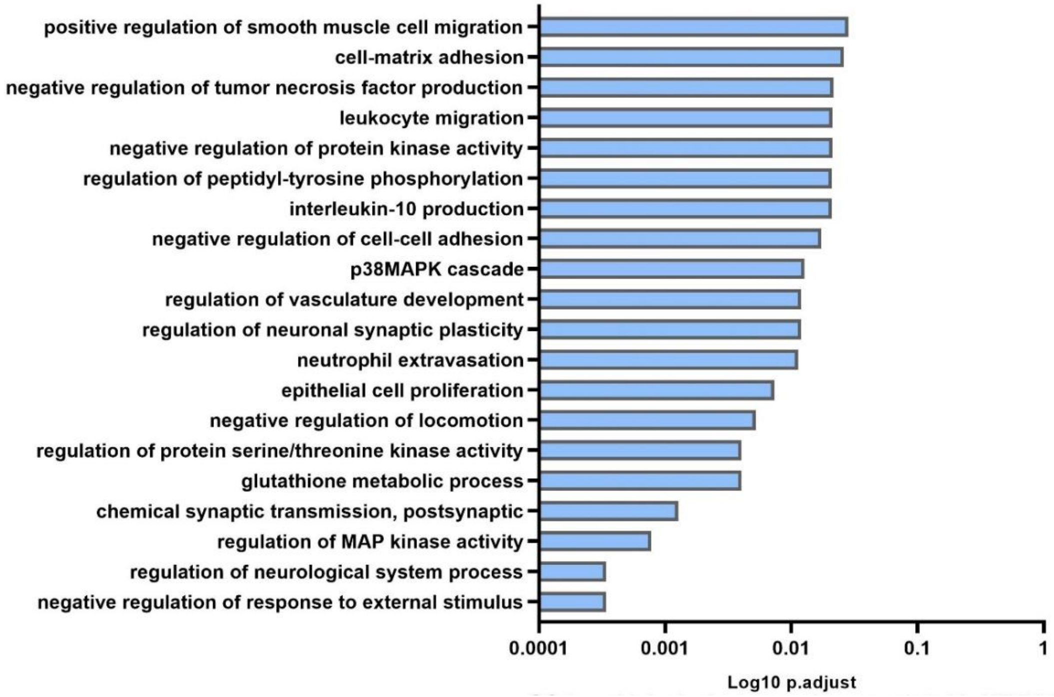

Supplementary Fig. 8. GO term biological process downregulated in WDR34 p.Gly394Ser clone in ciliated cells (The top 20 Gene Ontology (GO) terms are shown after similar terms have been removed. The full list of GO terms is provided in Supplementary Data 3).

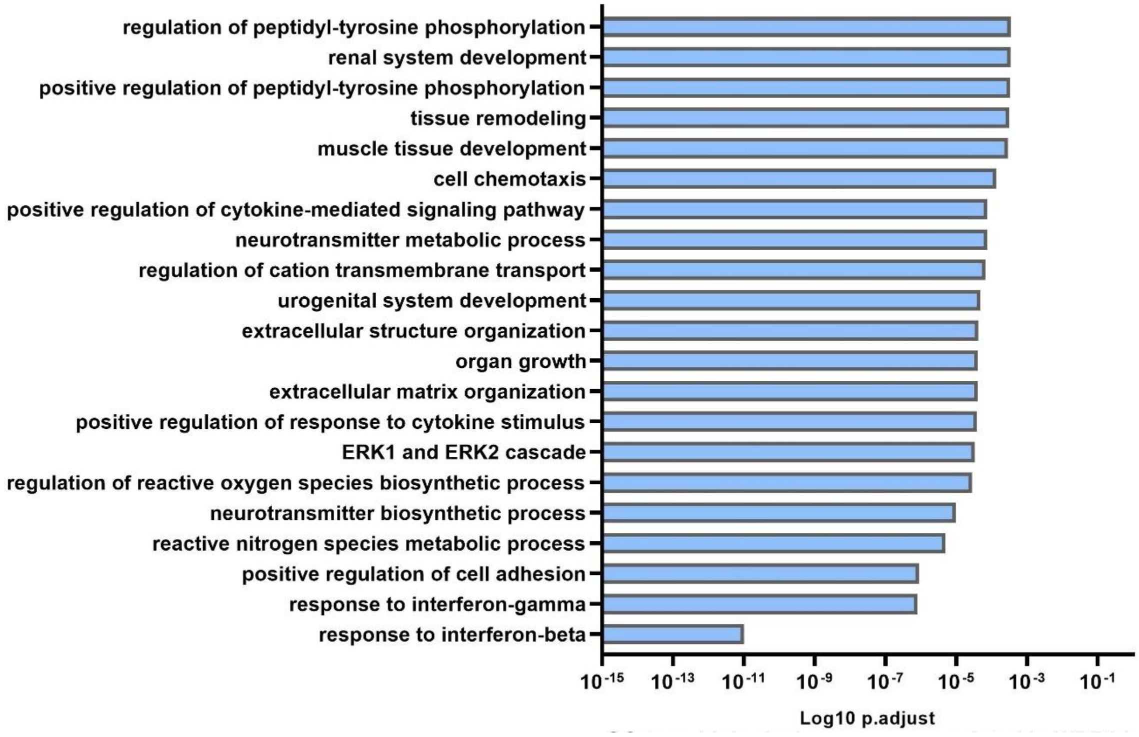

Supplementary Fig.9. GO term biological process upregulated in WDR34 p.Gly394Ser clone in ciliated cells (The top 20 Gene Ontology (GO) terms are shown after similar terms have been removed. The full list of GO terms is provided in Supplementary Data 3).

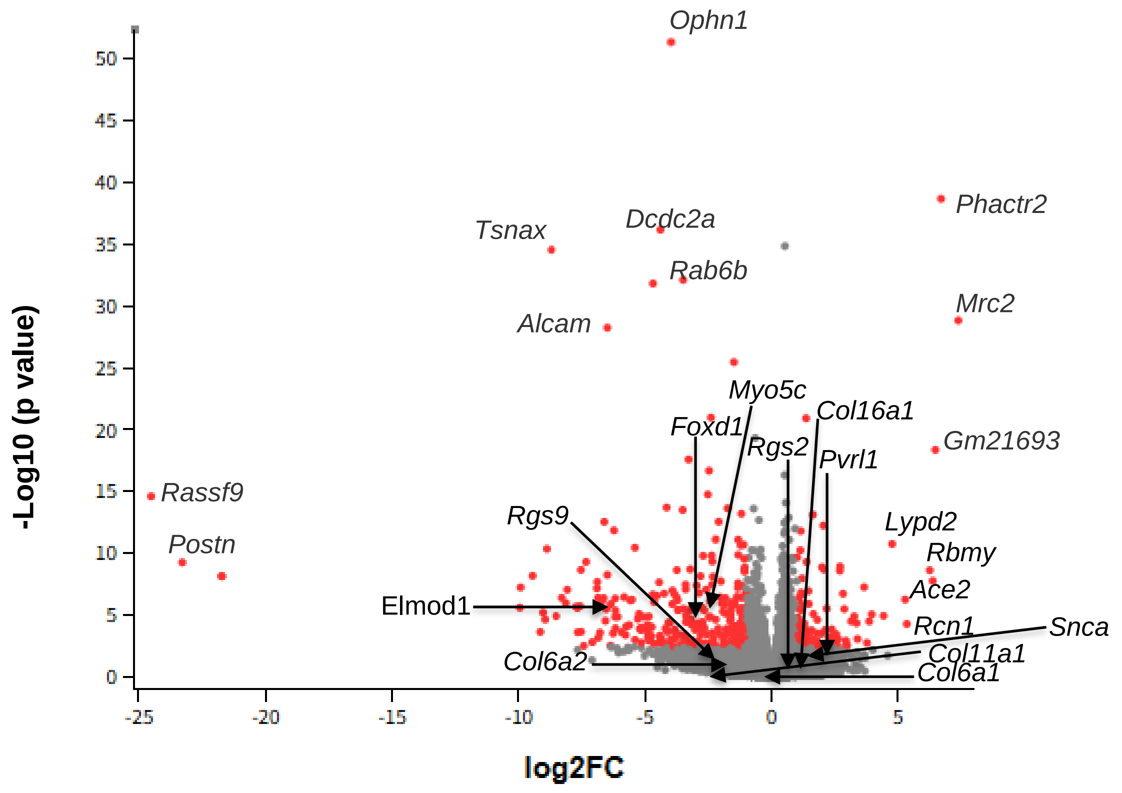

Supplementary Fig. 10. Volcano plot indicating differentially expressed genes in WDR60 p.Ala911Val clone versus controls in ciliated cells.

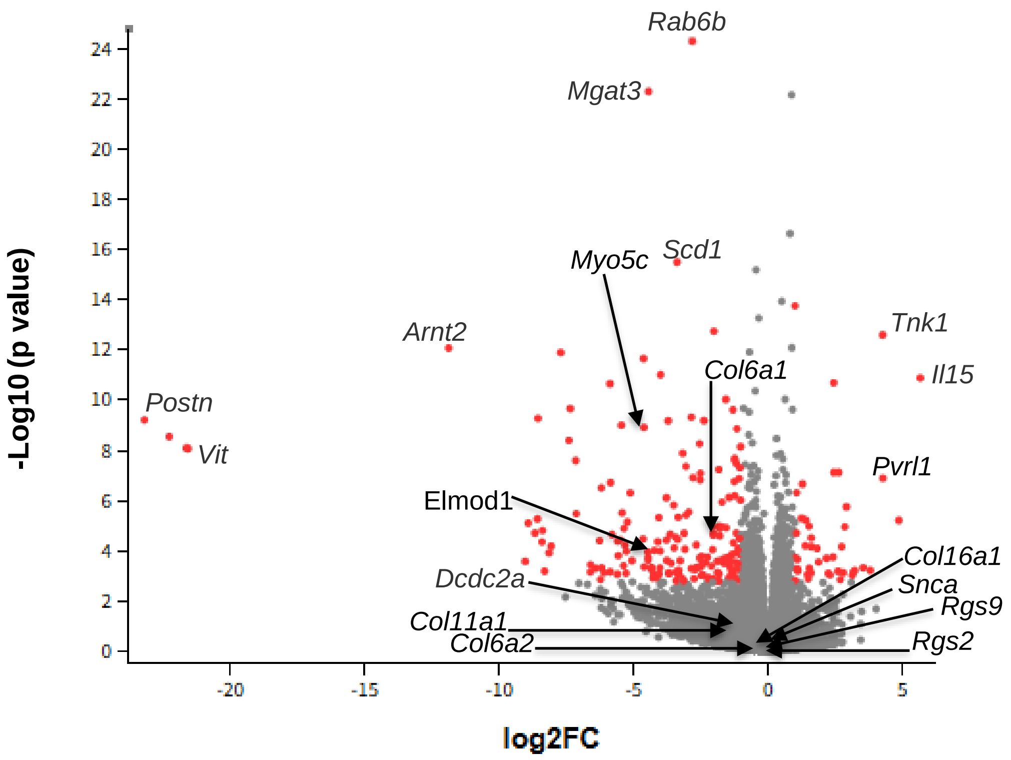

Supplementary Fig. 11. Volcano plot indicating differentially expressed genes in WDR34 p.Arg183Trp versus controls in ciliated cells.

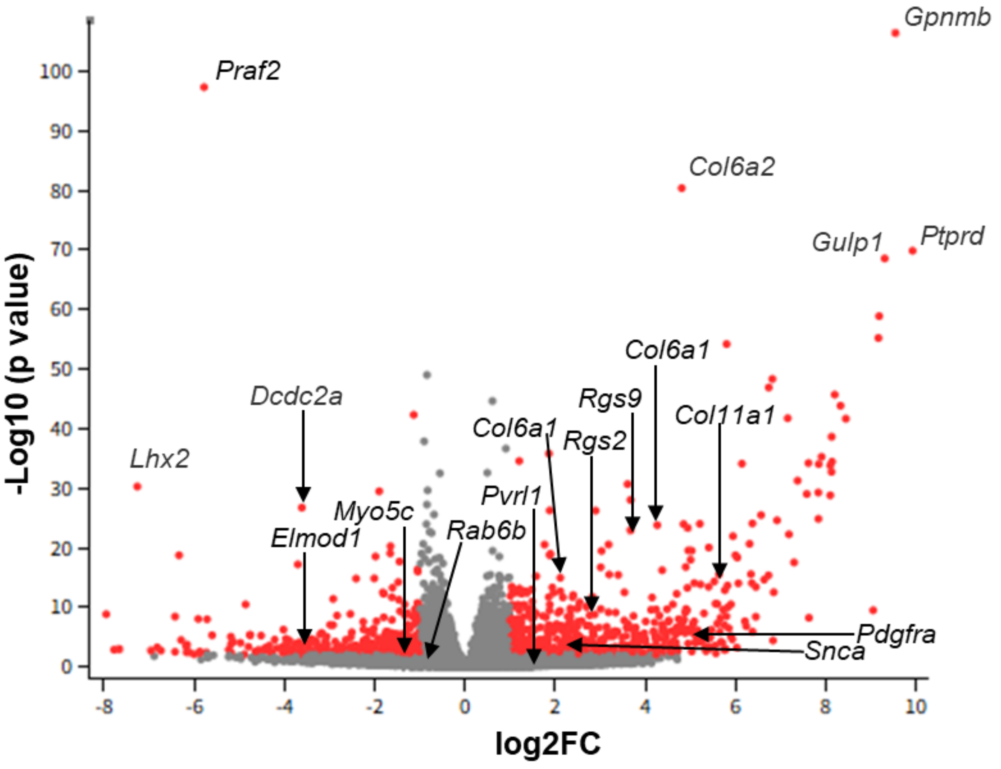

Supplementary Fig. 12. Volcano plot indicating differentially expressed genes in WDR34 p.Gly394Ser versus controls in ciliated cells.

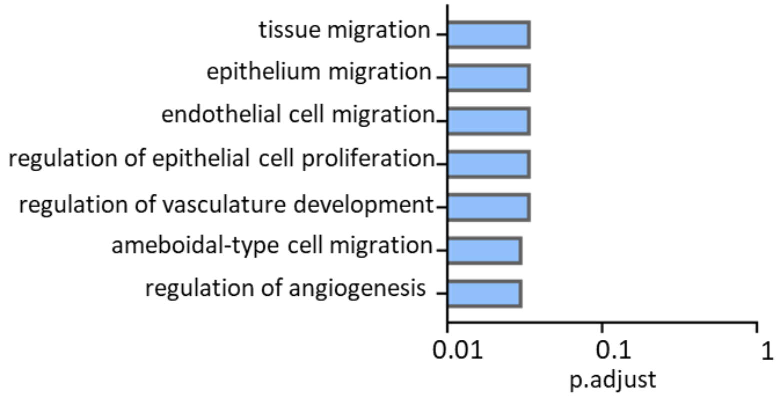

Supplementary Fig.13. GO term biological process downregulated in dynein-2 mutants versus controls, shared between ciliated and non-ciliated cells. p.adjust value is from ciliated cells RNA sequencing analysis.

I

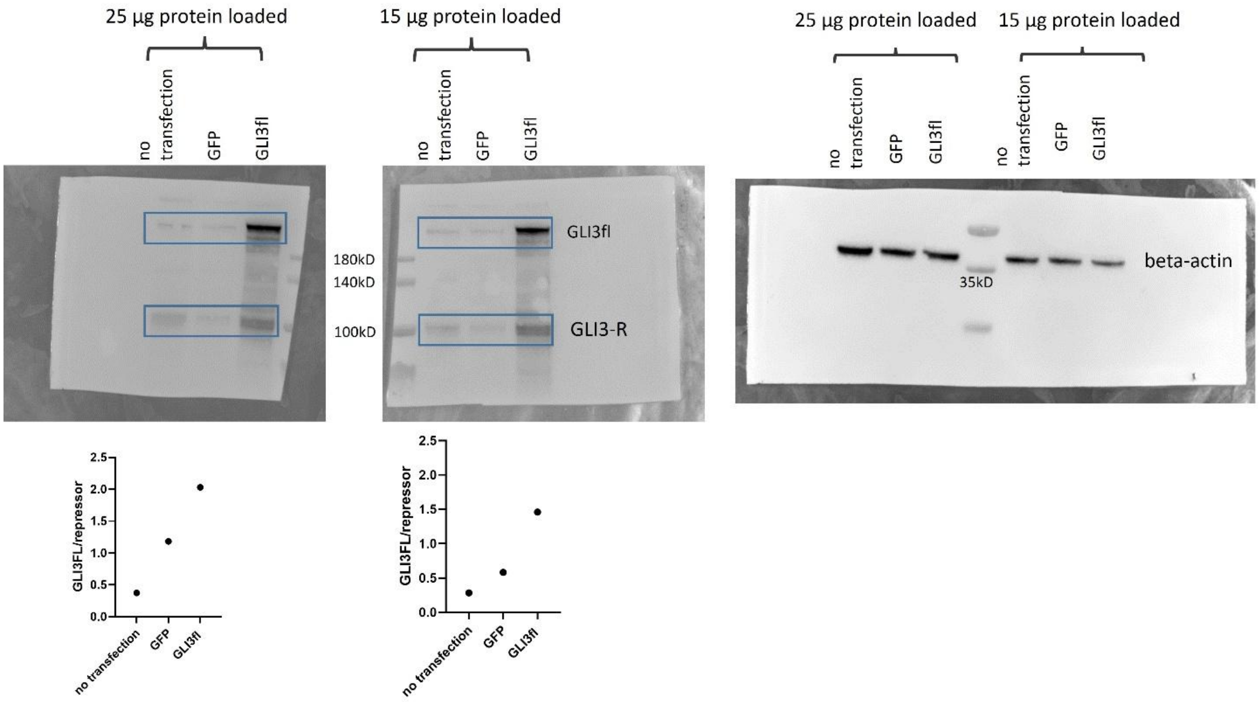

II

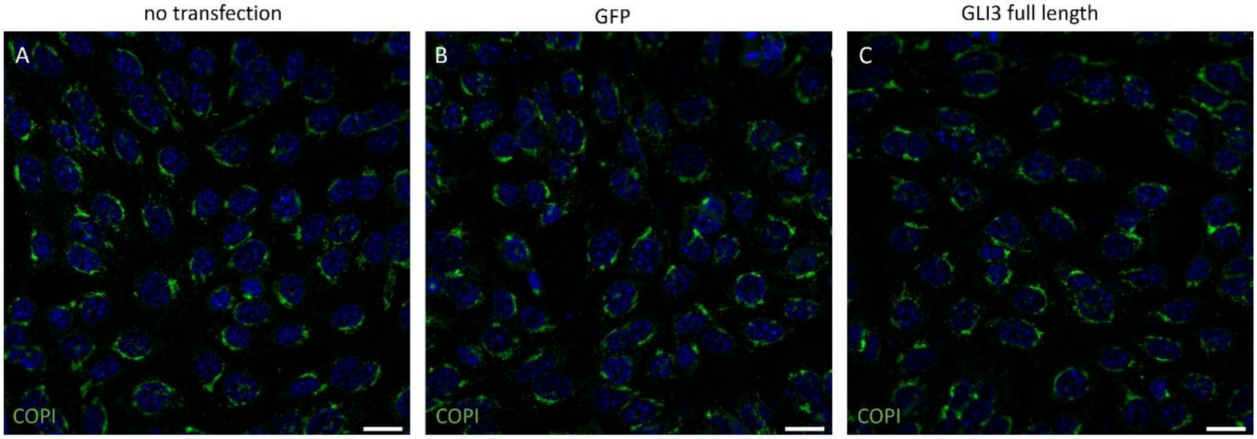

IMCD3 cells 24 hours post transfection with GFP (B), GLI3 full length (C) or no transfection (A). Scale bars 20 µm.

Supplementary Fig. 14. I) Western blot analysis indicating the enhanced expression of GLI3Fl in cells transfected with GLI3Fl plasmid compared to cells transfected with GFP or without any transfection. II) Immunofluorescence analysis of control cells overexpressing GLI3Fl shows no defect in COPI vesicles.
