# Supplementary material for: Base editing-derived models of human WDR34 and WDR60 disease alleles replicate retrograde intraflagellar transport (IFT) and hedgehog signaling defects
Source: Commun Biol. 2026 Jul 1;9:889. doi: 10.1038/s42003-026-10507-2 (PMC13328300; doi:10.1038/s42003-026-10507-2)
Supplement: Supplementary file 2 — Description of Additional Supplementary Files [file 42003_2026_10507_MOESM2_ESM.docx]

**Description of Additional Supplementary File**
File name: Supplementary data 1
Description: WDR60p.Ala911Val_ciliated cells GO_BP

File name: Supplementary data 2
Description: WDR34p.Arg183trp_ciliated cells GO BP

File name: Supplementary data 3
Description: WDR34p.Gly394Ser_ciliated cells GO_BP

File name: Supplementary data 4
Description: Differentially expressed genes WDR60p.Ala911Val_ciliated cells

File name: Supplementary data 5
Description: Differentially expressed genes WDR34p.Arg183trp_ciliated cells

File name: Supplementary data 6
Description: Differentially expressed genes WDR34p.Gly394Ser_ciliated cells

File name: Supplementary data 7
Description: Differentially expressed genes WDR34p.Gly394Ser_ciliated cells

File name: Supplementary data 8
Description: Raw data for Figure 4 F
