# Supplementary material for: Base editing-derived models of human WDR34 and WDR60 disease alleles replicate retrograde intraflagellar transport (IFT) and hedgehog signaling defects
Source: Commun Biol. 2026 Jul 1;9:889. doi: 10.1038/s42003-026-10507-2 (PMC13328300; doi:10.1038/s42003-026-10507-2)
Supplement: Supplementary file 11 — Reporting Summary [file 42003_2026_10507_MOESM11_ESM.pdf]

nature portfolio

Corresponding author(s): Miriam Schmidts

Last updated by author(s): 15.06.2026

Reporting Summary

Nature Portfolio wishes to improve the reproducibility of the work that we publish. This form provides structure for consistency and transparency in reporting. For further information on Nature Portfolio policies, see our Editorial Policies and the Editorial Policy Checklist.

Statistics

For all statistical analyses, confirm that the following items are present in the figure legend, table legend, main text, or Methods section.

n/a	Confirmed	
☐	☒	The exact sample size (*n*) for each experimental group/condition, given as a discrete number and unit of measurement
☐	☒	A statement on whether measurements were taken from distinct samples or whether the same sample was measured repeatedly
☐	☒	The statistical test(s) used AND whether they are one- or two-sided<br>*Only common tests should be described solely by name; describe more complex techniques in the Methods section.*
☒	☐	A description of all covariates tested
☐	☒	A description of any assumptions or corrections, such as tests of normality and adjustment for multiple comparisons
☐	☒	A full description of the statistical parameters including central tendency (e.g. means) or other basic estimates (e.g. regression coefficient) AND variation (e.g. standard deviation) or associated estimates of uncertainty (e.g. confidence intervals)
☐	☒	For null hypothesis testing, the test statistic (e.g. *F*, *t*, *r*) with confidence intervals, effect sizes, degrees of freedom and *P* value noted<br>*Give P values as exact values whenever suitable.*
☒	☐	For Bayesian analysis, information on the choice of priors and Markov chain Monte Carlo settings
☐	☐	For hierarchical and complex designs, identification of the appropriate level for tests and full reporting of outcomes
☐	☒	Estimates of effect sizes (e.g. Cohen's *d*, Pearson's *r*), indicating how they were calculated

Our web collection on statistics for biologists contains articles on many of the points above.

Software and code

Policy information about availability of computer code

Data collection	No software for data collection was used
Data analysis	Transcriptomics bioinformatics<br>RNA sequence analyses were performed in house as previously described (Tosic et al., 2019). In brief, sequence reads were mapped to the mouse reference genome GRCm38/mm10 (iGenomes, Illumina; chromosomes 1-19, X, Y, M) using the Rsubread v1.28.1 package in R v3.4.4. Gene counts were retrieved from the mapping, applying the Rsubread::featureCounts function with the iGenomes reference genome gtf file for annotation (archive-2015-07-17-32-40 and archive-2015-07-17-33-26 for GRCM38). For differential expression analysis, DESeq2 v1.18.1 was used. First, the counts were normalized by library size (DESeq2::estimateSizeFactors) and by gene-wise dispersion (gene-wise geometric mean over the samples; DESeq2::estimateDispersions), then, the differential expression analyses were performed applying negative binomial generalized linear model fitting and Wald statistics on the normalized count data (DESeq2::DESeq2). Subsequently, the results were filtered for adjusted p < 0.05 and the log2 fold change values (log2FC) indicated in the figure legends. Interactive volcano and MDS plots were generated using the R package Glimma (1.10.1) and heatmaps using pheatmap (v1.0.12). For GO term enrichment analyses, gene symbols were converted to gene IDs using biomaRt (v2.38.0) and subsequently, enrichment was assessed and visualized using GO.db (3.7.0) and clusterProfiler (3.10.1).

For manuscripts utilizing custom algorithms or software that are central to the research but not yet described in published literature, software must be made available to editors and reviewers. We strongly encourage code deposition in a community repository (e.g. GitHub). See the Nature Portfolio guidelines for submitting code & software for further information.

Data

Policy information about availability of data

All manuscripts must include a data availability statement. This statement should provide the following information, where applicable:

- Accession codes, unique identifiers, or web links for publicly available datasets
- A description of any restrictions on data availability
- For clinical datasets or third party data, please ensure that the statement adheres to our policy

Transcriptomic data is accessivle in GEO, accession number GSE208712

Research involving human participants, their data, or biological material

Policy information about studies with human participants or human data. See also policy information about sex, gender (identity/presentation), and sexual orientation and race, ethnicity and racism.

Reporting on sex and gender	no sex and gender bases analyses were performed however sex of included patients is reported with consent of the faniily. Sex assignment is based on clinical opinion of the referring clinican and self reporting and refers to biological sex
Reporting on race, ethnicity, or other socially relevant groupings	no analyses bases on race, ethnicity or socially relevant groupings were performed, however country of origin is stated in the mansucript as reported by the including clinician. Consent for this was obtained from the fmaily
Population characteristics	No population based analyses were performed
Recruitment	Referring clinician referred the patient and parents to idnetify the underlying genetic cause of the patient`s health problems (genetic diagnsotics)
Ethics oversight	Molecular genetic diagnsotics was performed under the Innovative Diagnostics Programme Radboudumc Nijmegen, The Netehrlands, ethics commitee Arnhem Nijmegen and Freiburg University Ethics commitee, Feiburg, Germany under EKFR 122/20 Project P1

Note that full information on the approval of the study protocol must also be provided in the manuscript.

Field-specific reporting

Please select the one below that is the best fit for your research. If you are not sure, read the appropriate sections before making your selection.

☒ Life sciences ☐ Behavioural & social sciences ☐ Ecological, evolutionary & environmental sciences

For a reference copy of the document with all sections, see nature.com/documents/nr-reporting-summary-flat.pdf

Life sciences study design

All studies must disclose on these points even when the disclosure is negative.

Sample size	This study is not a clinical study nor a biostatistics study. There are few statistical analyses pefofrmed e.g. regarding ciliated cells mutant vs wildtype, cilia length, IFT accumualtion or wetsren blot quantification. n= 03 biological replicates were used as minimal datasets per genotypes
Data exclusions	no data was excluded
Replication	n= 3 biological replicates were used as minimal approach
Randomization	no randomization was performed
Blinding	no

Reporting for specific materials, systems and methods

We require information from authors about some types of materials, experimental systems and methods used in many studies. Here, indicate whether each material, system or method listed is relevant to your study. If you are not sure if a list item applies to your research, read the appropriate section before selecting a response.

Materials & experimental systems

n/a	Involved in the study
☐	☒ Antibodies
☐	☒ Eukaryotic cell lines
☒	☐ Palaeontology and archaeology
☒	☐ Animals and other organisms
☐	☒ Clinical data
☒	☐ Dual use research of concern
☒	☐ Plants

Methods

n/a	Involved in the study
☐	☐ ChIP-seq
☐	☐ Flow cytometry
☐	☐ MRI-based neuroimaging

Antibodies

Antibodies used

All antibodies are shown in the material and methods section: rabbit polyclonal IFT88 (13967-1-AP, Proteintech, Rosemont, IL, USA) , mouse monoclonal gamma tubulin primary antibodies (T6557, Sigma, Taufkirchen, Germany), Alexa fluor 568 goat anti-rabbit (A11011, Life technologies, Carlsbad, CA, USA) and Alexa fluor 633 goat anti-mouse IgG1 (A21126, Life technologies, Carlsbad, CA, USA) , goat polyclonal GLI3 antibody (AF3690, R&D systems, Minneapolis, Canada). , mouse monoclonal aPVRL1 (MABT61, Sigma, Taufkirchen, Germany), Rabbit polyclonal beta-actin antibody was (Ab8227, Abcam, Cambridge, UK)

Validation

Antibody validation can be retrieved from suppliers homepages

Eukaryotic cell lines

Policy information about cell lines and Sex and Gender in Research

Cell line source(s)

IMCD3 cells were a kind gift if Prof Maxence nachury, Stanford University

Authentication

none

Mycoplasma contamination

none

Commonly misidentified lines
(See ICLAC register)

n/a

Clinical data

Policy information about clinical studies

All manuscripts should comply with the ICMJE guidelines for publication of clinical research and a completed CONSORT checklist must be included with all submissions.

Clinical trial registration

N/a, not a clinical trial but genetic diagnosis performed in a single family, ethics approval from radboudumc Nijmegen and EKFR 122/20

Study protocol

Exome sequencing of children with unclear underlying molecualr cause of skeleto-renal syndromic phneotypes

Data collection

n/a not a clinical trial

Outcomes

n/a not a clinical trial

Plants

Seed stocks

n/a

Novel plant genotypes

n/a

Authentication

n/a

ChIP-seq

Data deposition

☐ Confirm that both raw and final processed data have been deposited in a public database such as GEO.

☐ Confirm that you have deposited or provided access to graph files (e.g. BED files) for the called peaks.

Data access links<br>*May remain private before publication.*	n/a no chipseq perforemd, trnascriptomics data is deposited under GEO GSE168582
Files in database submission	n/a
Genome browser session<br>(e.g. UCSC)	n/a

Methodology

Replicates	n/a
Sequencing depth	n/a
Antibodies	n/a
Peak calling parameters	n/a
Data quality	n/a
Software	n/a

Flow Cytometry

Plots

Confirm that:

☐ The axis labels state the marker and fluorochrome used (e.g. CD4-FITC).

☐ The axis scales are clearly visible. Include numbers along axes only for bottom left plot of group (a 'group' is an analysis of identical markers).

☐ All plots are contour plots with outliers or pseudocolor plots.

☐ A numerical value for number of cells or percentage (with statistics) is provided.

Methodology

Sample preparation	*Describe the sample preparation, detailing the biological source of the cells and any tissue processing steps used.*
Instrument	*Identify the instrument used for data collection, specifying make and model number.*
Software	*Describe the software used to collect and analyze the flow cytometry data. For custom code that has been deposited into a community repository, provide accession details.*
Cell population abundance	*Describe the abundance of the relevant cell populations within post-sort fractions, providing details on the purity of the samples and how it was determined.*
Gating strategy	*Describe the gating strategy used for all relevant experiments, specifying the preliminary FSC/SSC gates of the starting cell population, indicating where boundaries between "positive" and "negative" staining cell populations are defined.*

☐ Tick this box to confirm that a figure exemplifying the gating strategy is provided in the Supplementary Information.

Magnetic resonance imaging

Experimental design

Design type	*Indicate task or resting state; event-related or block design.*
Design specifications	*Specify the number of blocks, trials or experimental units per session and/or subject, and specify the length of each trial or block (if trials are blocked) and interval between trials.*

Behavioral performance measures | *State number and/or type of variables recorded (e.g. correct button press, response time) and what statistics were used to establish that the subjects were performing the task as expected (e.g. mean, range, and/or standard deviation across subjects).*

Acquisition

Imaging type(s) | *Specify: functional, structural, diffusion, perfusion.*

Field strength | *Specify in Tesla*

Sequence & imaging parameters | *Specify the pulse sequence type (gradient echo, spin echo, etc.), imaging type (EPI, spiral, etc.), field of view, matrix size, slice thickness, orientation and TE/TR/flip angle.*

Area of acquisition | *State whether a whole brain scan was used OR define the area of acquisition, describing how the region was determined.*

Diffusion MRI ☐ Used ☐ Not used

Preprocessing

Preprocessing software | *Provide detail on software version and revision number and on specific parameters (model/functions, brain extraction, segmentation, smoothing kernel size, etc.).*

Normalization | *If data were normalized/standardized, describe the approach(es): specify linear or non-linear and define image types used for transformation OR indicate that data were not normalized and explain rationale for lack of normalization.*

Normalization template | *Describe the template used for normalization/transformation, specifying subject space or group standardized space (e.g. original Talairach, MNI305, ICBM152) OR indicate that the data were not normalized.*

Noise and artifact removal | *Describe your procedure(s) for artifact and structured noise removal, specifying motion parameters, tissue signals and physiological signals (heart rate, respiration).*

Volume censoring | *Define your software and/or method and criteria for volume censoring, and state the extent of such censoring.*

Statistical modeling & inference

Model type and settings | *Specify type (mass univariate, multivariate, RSA, predictive, etc.) and describe essential details of the model at the first and second levels (e.g. fixed, random or mixed effects; drift or auto-correlation).*

Effect(s) tested | *Define precise effect in terms of the task or stimulus conditions instead of psychological concepts and indicate whether ANOVA or factorial designs were used.*

Specify type of analysis: ☐ Whole brain ☐ ROI-based ☐ Both

Statistic type for inference | *Specify voxel-wise or cluster-wise and report all relevant parameters for cluster-wise methods.*

(See Eklund et al. 2016)

Correction | *Describe the type of correction and how it is obtained for multiple comparisons (e.g. FWE, FDR, permutation or Monte Carlo).*

Models & analysis

n/a	Involved in the study
☒	☐ Functional and/or effective connectivity
☒	☐ Graph analysis
☒	☐ Multivariate modeling or predictive analysis
